# Supplementary material for: Risk of preterm birth associated with maternal gingival inflammation and oral hygiene behaviours in rural Nepal: a community-based, prospective cohort study
Source: BMJ Open. 2020 Aug 20;10(8):e036515. doi: 10.1136/bmjopen-2019-036515 (PMC7443267; doi:10.1136/bmjopen-2019-036515)
Supplement: Author's manuscript [file bmjopen-2019-036515.draft_revisions.pdf]

BMJ Open is committed to open peer review. As part of this commitment we make the peer review history of every article we publish publicly available.

When an article is published we post the peer reviewers' comments and the authors' responses online. We also post the versions of the paper that were used during peer review. These are the versions that the peer review comments apply to.

The versions of the paper that follow are the versions that were submitted during the peer review process. They are not the versions of record or the final published versions. They should not be cited or distributed as the published version of this manuscript.

BMJ Open is an open access journal and the full, final, typeset and author-corrected version of record of the manuscript is available on our site with no access controls, subscription charges or pay-per-view fees (http://bmjopen.bmj.com).

If you have any questions on BMJ Open's open peer review process please email info.bmjopen@bmj.com

BMJ Open

Risk of preterm birth associated with maternal gingival inflammation and oral hygiene behaviors in rural Nepal

Journal:	*BMJ Open*
Manuscript ID	bmjopen-2019-036515
Article Type:	Original research
Date Submitted by the Author:	20-Dec-2019
Complete List of Authors:	Erchick, Daniel; Johns Hopkins University Bloomberg School of Public Health, International Health<br>Khatry, Subarna; Nepal Nutrition Intervention Project<br>Agrawal, Nitin; Institute of Medicine, Tribhuhvan University, Department of Dentistry<br>Katz, Joanne; Johns Hopkins University Bloomberg School of Public Health, International Health<br>LeClerq, Steven; Johns Hopkins University Bloomberg School of Public Health, International Health; Nepal Nutrition Intervention Project Sarlahi<br>Rai, Bhola; Nepal Nutrition Intervention Project Sarlahi<br>Reynolds, Mark; University of Maryland School of Dentistry, Department of Advanced Oral Sciences and Therapeutics<br>Mullany, Luke C.; Johns Hopkins University Bloomberg School of Public Health, International Health
Keywords:	EPIDEMIOLOGY, OBSTETRICS, ORAL MEDICINE, Community child health < PAEDIATRICS, PERINATOLOGY, PUBLIC HEALTH

SCHOLARONE™
Manuscripts

I, the Submitting Author has the right to grant and does grant on behalf of all authors of the Work (as defined in the below author licence), an exclusive licence and/or a non-exclusive licence for contributions from authors who are: i) UK Crown employees; ii) where BMJ has agreed a CC-BY licence shall apply, and/or iii) in accordance with the terms applicable for US Federal Government officers or employees acting as part of their official duties; on a worldwide, perpetual, irrevocable, royalty-free basis to BMJ Publishing Group Ltd ("BMJ") its licensees and where the relevant Journal is co-owned by BMJ to the co-owners of the Journal, to publish the Work in this journal and any other BMJ products and to exploit all rights, as set out in our licence.

The Submitting Author accepts and understands that any supply made under these terms is made by BMJ to the Submitting Author unless you are acting as an employee on behalf of your employer or a postgraduate student of an affiliated institution which is paying any applicable article publishing charge ("APC") for Open Access articles. Where the Submitting Author wishes to make the Work available on an Open Access basis (and intends to pay the relevant APC), the terms of reuse of such Open Access shall be governed by a Creative Commons licence – details of these licences and which Creative Commons licence will apply to this Work are set out in our licence referred to above.

Other than as permitted in any relevant BMJ Author's Self Archiving Policies, I confirm this Work has not been accepted for publication elsewhere, is not being considered for publication elsewhere and does not duplicate material already published. I confirm all authors consent to publication of this Work and authorise the granting of this licence.

For peer review only

Risk of preterm birth associated with maternal gingival inflammation and oral hygiene behaviors in rural Nepal

Daniel J. Erchick[a], PhD, MPH, Subarna K. Khatry[b], MBBS, Nitin K. Agrawal[c], MDS, MSc, Joanne Katz[a], ScD, MS, Steven C. LeClerq[a,b], MPH, Bhola Rai[b], Mark A. Reynolds[d], DDS, PhD, MA, Luke C. Mullany[a], PhD, MHS, MS

Affiliations:

[a]Department of International Health, Johns Hopkins Bloomberg School of Public Health, Baltimore, MD, United States

[b]Nepal Nutrition Intervention Project – Sarlahi (NNIPS), Krishna Galli, Lalitpur, Kathmandu, Nepal

[c]Department of Dentistry, Institute of Medicine, Tribhuhvan University. Kathmandu, Nepal

[d]Department of Advanced Oral Sciences and Therapeutics, University of Maryland School of Dentistry, Baltimore, MD, United States

Emails: Khatry SK (skhatry@wlink.com.np), Agrawal NK (cdrnitin@gmail.com), Katz J (jkatz1@jhu.edu), LeClerq SC (sleclerq@mos.com.np), Rai B (bholarai19@gmail.com), Reynolds MA (MReynolds@umaryland.edu), Mullany LC (lmullany@jhu.edu)

Correspondence: Daniel J. Erchick, PhD, MPH. Department of International Health, Johns Hopkins Bloomberg School of Public Health, 615 N. Wolfe Street, Baltimore, MD, 21205, United States. derchick@jhu.edu

Word Count: 3,751

For peer review only

ABSTRACT

Objectives: Observational studies have identified associations between periodontitis and adverse pregnancy outcomes, but randomized controlled trials evaluating the efficacy of periodontal therapy have yielded inconsistent results. Few studies have explored relationships between gingival inflammation and these outcomes or been conducted in rural, low-income communities, where confounding risk factors differ from other settings.

Methods: We conducted a community-based, prospective cohort study to estimate associations between severity of gingival inflammation and preterm birth among pregnant women (<26 weeks gestation) in rural Nepal. Our primary exposure was gingival inflammation, defined as bleeding on probing (BOP) ≥10%, stratified by severity as BOP <30% and BOP ≥30%. A secondary exposure, mild periodontitis, was defined as ≥2 interproximal sites with probing depth (PD) ≥4 mm (different teeth) or one site with PD≥5 mm. The primary outcome was preterm birth (<37 weeks gestation). Poisson regression was used to model this relationship, adjusting for potential confounders.

Results: Of 1,394 women, 554 (39.7%) had gingival inflammation, 54 (3.9%) mild periodontitis, and 197 (14.1%) delivered preterm. In the adjusted regression model, increasing severity of gingival inflammation was associated with a non-significant increase in risk of preterm birth (BOP ≥30% vs. no BOP: aRR 1.37, 95% CI: 0.81, 2.32). A secondary analysis, stratifying participants by when in pregnancy their oral health status was assessed, showed an association between gingival inflammation and preterm birth among women examined in their first trimester

For peer review only - http://bmjopen.bmj.com/site/about/guidelines.xhtml
For peer review only

(BOP ≥30% vs. no BOP: aRR 2.57, 95% CI: 1.11, 5.95), but not later in pregnancy (BOP ≥30% vs. no BOP: aRR 1.05, 95% CI: 0.52, 2.11).

Conclusions: Gingival inflammation among women examined early in pregnancy and poor oral hygiene behaviors were risk factors for preterm birth. Future studies should evaluate the effect of community-based oral health interventions that specifically target gingival inflammation, delivered early or before pregnancy, on preterm birth.

Strengths and Limitations of this Study

- This study collected data through a large, prospective, community-based cohort in a rural, low-resource setting.
- The study population had low prevalence of known confounders of the relationship of interest, which are common in other populations.
- Some clinical periodontal measures were not collected due to visit time constraints, for example, recession on interproximal sites or gingival index score.
- Preterm birth classification was based upon maternal self-report of last menstrual period (LMP) instead of gold standard ultrasound examination.

For peer review only

For peer review only - http://bmjopen.bmj.com/site/about/guidelines.xhtml

INTRODUCTION

Annually, 2.5 million babies die prior to 28 days of life, and preterm birth is the leading cause of these deaths.[1] Preterm newborns that survive are at substantial risk of mortality from other causes, long-term disabilities such as neurological and developmental impairments, and non-communicable diseases.[2] In low- and middle-income countries (LMICs), where the majority of preterm births occur, therapeutic interventions are often unavailable and difficult to scale up, especially in communities where many mothers deliver at home or in primary facilities without skilled care (e.g. South Asia).[3]

Periodontal disease includes several inflammatory conditions, typically initiated by oral bacteria, beginning with reversible accumulation of plaque and inflammation of gingival tissue (gingivitis) and progressing to irreversible breakdown of the supportive tissues of the teeth and tooth loss (periodontitis).[4] Onset of new and worsening of existing gingival inflammation during pregnancy are normal and well documented, peaking in the second or third trimester.[5, 6] Major physiological and hormonal changes occur during pregnancy with wide ranging effects on the body, including increased permeability of gingival capillaries, altered immune system activity, and shifts in composition of the sub-gingival microbiome, including proliferation of aggressive bacteria associated with periodontitis, such as *Porphyromonas gingivalis.*[7, 8]

Periodontal disease in pregnant women has been associated with preterm birth and other adverse pregnancy outcomes.[9-11] Randomized controlled trials (RCTs) evaluating the impact of periodontal therapy during pregnancy on adverse pregnancy outcomes have produced inconsistent results.[9, 12-18] One meta-analysis found a significant effect of periodontal therapy

For peer review only - http://bmjopen.bmj.com/site/about/guidelines.xhtml

among women at high risk of preterm birth.[19] Although the mechanisms underlying the observed association are unclear, hypotheses include hematogenic translocation of periodontal pathogens or their byproducts to the fetal-placental unit, or action of inflammatory mediators in the periodontium on levels of systemic inflammation.[20] Alternatively, the observed relationship could be due to a common confounding factor, such as a genetic hyper-inflammatory phenotype, responsible for both increased risk for periodontal disease and adverse pregnancy outcomes.[21]

Few studies have evaluated whether the extent or severity of gingival inflammation are associated with risk of preterm birth or other adverse pregnancy outcomes. Further, studies of this association have been nearly universally facility-based, whether in high- or low-income settings. Understanding this relationship from a population-based perspective in low resource communities can offer certain benefits from an epidemiological perspective. Many populations in LMICs have lower prevalence of important confounding factors of this relationship, such as smoking, alcohol use, and chronic diseases (e.g. hypertension or diabetes). Community-based studies can avoid selection bias associated with hospital-based studies, particularly in populations where home delivery remains common, as is the case in much of South Asia. Given these potential benefits, we conducted a community-based, prospective cohort study to estimate the association between gingival inflammation and preterm birth among women in a rural community in the terai (plains) region of Nepal.

For peer review only

For peer review only - http://bmjopen.bmj.com/site/about/guidelines.xhtml

METHODS

We enrolled pregnant women <26 weeks gestation in a community-based, prospective cohort study of maternal gingival inflammation and adverse pregnancy outcomes across nine village development committees (VDCs) in Sarlahi District, Nepal, between January and November 2016. Participants in this study were identified and determined eligible using the infrastructure of a large community-based randomized trial of topical applications for newborn massage, the Nepal Oil Massage Study (NOMS) (NCT01177111), which was actively enrolling a population-based sample of pregnant women in this study area.

Study visits

Study visits were conducted in participant homes because of the wide dispersion of households across this rural community and the impracticality of bringing participants to a central location. Data on participant demographics, vital signs and morbidities during pregnancy, oral hygiene practices, care-seeking, and knowledge, and other characteristics were collected through a series of questionnaires administered over several visits during the course of pregnancy. Data collection teams were notified of the birth outcome by a locally resident study staff member, and the date of birth and other data concerning the mother and newborn were collected as soon as possible after delivery.

Oral health examinations were performed by five auxiliary nurse-midwives who were trained as "community-based oral health workers." Their training lasted 3-4 weeks and included classroom instruction and practice of periodontal techniques conducted by an experienced dentist (NKA) at the Department of Dentistry, Institute of Medicine, Tribhuvann University, Kathmandu, Nepal.

For peer review only - http://bmjopen.bmj.com/site/about/guidelines.xhtml

We estimated the validity of PD measurements of the oral health workers relative to the dentist (NKA), finding percent agreement, weighted kappa scores, and intraclass correlation coefficients, with an allowance of PD ±1 mm, exceeded 99%, 0.7, and 0.9, respectively.

Periodontal measurements

Oral health workers used portable dental equipment to conduct a full mouth examination. Periodontal measurements were made using a color Williams probe (Hu-Friedy, Chicago, IL, USA). PD was measured on six sites per tooth (disto-, mid-, and mesial- aspects of buccal and lingual surfaces) and CEJ-GM distance on two sites per tooth (mid- buccal and lingual aspects), excluding third molars. Presence or absence of BOP was recorded for any buccal or lingual probing site of each tooth. PD values were recorded in millimeters rounded to the next higher whole number. CEJ-GM distances were recorded similarly. If the free gingiva was coronal to the CEJ, the CEJ-GM measurement was recorded as 0. Clinical attachment loss (CAL) was calculated by summing PD and CEJ-GM distances.

Outcome/exposure definitions

Our primary exposure definition, gingival inflammation, was defined according to a classification scheme developed by the American Academy of Periodontology (AAP) and European Federation of Periodontology to update the 1999 classification of periodontal diseases and conditions.[22] Clinical health was defined as all sites PD ≤3 mm and BOP <10%.[23, 24] Gingival inflammation was defined as BOP ≥10%, stratified by severity as localized gingival inflammation (BOP 10%-30%) and generalized gingival inflammation (BOP ≥30%).[23-28]

For peer review only - http://bmjopen.bmj.com/site/about/guidelines.xhtml

We defined secondary exposure definitions for mild and moderate periodontitis based upon a modified version of Centers for Disease Control and AAP updated 2012 case definitions for population-based surveillance of periodontitis.[27, 29] Mild periodontitis was defined as ≥2 interproximal sites with PD≥4 mm (not on the same tooth) or one site with PD≥5 mm. Moderate periodontitis was defined as ≥2 interproximal sites with PD≥5 mm (not on the same tooth). CAL was not included in these definitions as we did not collect this measure at interproximal sites to shorten the visit time; however, clinical attachment level and probing depth are widely considered equivalent measures of periodontitis in younger adults.[30]

Gestational age was calculated using the last menstrual period (LMP) method as recalled by the mother at 5-weekly pregnancy surveillance home visits. Our 5-weekly pregnancy surveillance approach has several benefits, including short recall (≤5 weeks) and pregnancy testing, as compared to traditional LMP approaches, which often rely on LMP recall later in pregnancy or at time of delivery. Preterm birth was defined as a live birth or stillbirth <37 completed weeks of gestation at the time of delivery.

Statistical analysis

Bivariate analyses between participant characteristics and the outcome, preterm birth, were evaluated using t-tests and Poisson regression for continuous and binary/categorical variables, respectively. We calculated unadjusted and adjusted relative risks (RR & aRR) of preterm birth and associated 95% confidence internals (CI) using Poisson regression with robust variance, which was used due to occurrence of convergence issues with other regression methods. Given our sample size (n=1,394) and assuming a power of 80%, type I error of 0.05, and our pre-study

For peer review only - http://bmjopen.bmj.com/site/about/guidelines.xhtml

estimates of population prevalence of preterm birth (17%) and gingival inflammation (40%), we estimated that we could detect a relative risk of preterm birth of roughly >1.5.

Multivariable models were constructed by sequentially adding groups of covariates, beginning with maternal characteristics, oral hygiene behaviors, and socioeconomic factors. Covariates associated with preterm birth at the $p<0.10$ level in bivariate analyses were considered in these regression models. Additional variables, known through previous studies to be confounders of the periodontal disease and preterm birth relationship, were also included in regression models (including age, ethnicity, body mass index (BMI), primiparity, multiple births, and socioeconomic variables (i.e., literacy, education, and indicators of household wealth).

In an effort to remove a possibly attenuating effect of including cases of transient pregnancy-induced gingival inflammation, we examined the relationship stratified by timing of oral exam. Women were categorized into two trimester groups (<13 weeks and ≥13 to <26 weeks gestation). Similar to the primary analysis, for each trimester group we conducted adjusted relative risks (RR & aRR) of preterm birth and associated 95% confidence internals (CI) using Poisson regression with robust variance.

All statistical analyses were performed in STATA 14.2 (StataCorp, College Station, TX, USA). This study received ethical approval from the Institutional Review Board at Johns Hopkins Bloomberg School of Public Health (Baltimore, USA) and the Ethical Review Board of the Nepal Health Research Council (Kathmandu, Nepal). Patients or the public were not involved in the design, or conduct, or reporting, or dissemination plans of our research.

For peer review only - http://bmjopen.bmj.com/site/about/guidelines.xhtml

RESULTS

Participant characteristics

Between January 11, 2016, and November 26, 2016, among 2,821 pregnancies in the study area, 2,291 (81.2%) participants were identified by the parent trial as eligible for enrollment (<26 weeks gestation) (Figure 1). Among eligible participants, 1,478 (64.5%) were enrolled and six refused participation (0.3%). Another 807 (35.2%) were not visited for the purposes of the study because the eligible participants in the study area initially exceeded the capacity of the oral health workers to enroll women before their gestational age exceeded the exclusion criterion of <26 weeks. For logistical reasons, enrollment of women earlier in pregnancy was prioritized at the start of study enrollment, while enrollment of women later in pregnancy was prioritized towards the end of study enrollment. However, among eligible women, the included and excluded participant groups did not differ by age, education, or socio-economic factors.

Birth outcomes for the 1,474 women followed until the end of pregnancy were recorded as 1,345 single live births, 14 twin live births, 33 single stillbirths, 1 twin stillbirth, 1 set of twins where 1 was live born and 1 stillborn, 74 miscarriages, and 6 abortions. As our primary outcome was preterm birth, pregnancy was selected as the unit of analysis (and, therefore, twins were counted only once). There were 1,394 total pregnancies for analysis, of which 197 (14.1%) were preterm. Preterm deliveries included 163 (11.7%) moderate preterm (<37 to ≥32 weeks), 27 (1.9%) very preterm (<32 to ≥28 weeks), and 7 (0.5%) extremely preterm (<28 weeks). Over three-quarters of participants (n=1,053, 75.5%) were full term (≥37 weeks) and 144 (10.3%) were post-term (≥42 weeks).

For peer review only - http://bmjopen.bmj.com/site/about/guidelines.xhtml

Baseline demographic, periodontal, oral hygiene, and dental health care seeking characteristics of participants by severity of gingival inflammation were previously reported.[31] At enrollment, mean gestation was 14.5 (SD: 4.3) weeks (range: 6.4 to 27.7 weeks), with 574 women in their first trimester and 820 in their second. Participant age averaged 23.0 ± 4.6 years, and risk of preterm birth was higher (RR 1.93, CI: 1.01, 3.72) for women ≥35 years relative to those 18-35 years (Table 1). Several known confounders of the gingival inflammation and preterm birth relationship had a reported prevalence near 0% in this study population, including smoking and other tobacco use, alcohol use, and hypertension.

For peer review only

For peer review only - http://bmjopen.bmj.com/site/about/guidelines.xhtml

Table 1: Participant characteristics at enrollment (n=1,394)

Characteristic	All	Term	Preterm	Mean difference / RR (95% CI)*
**Age**				
Year (mean ± SD)	23.0 ± 4.6	23.0 ± 4.6	23.0 ± 5.1	0.02 (-0.68, 0.72)
**Age (years)**				
<18	151 (10.8)	124 (10.4)	27 (13.7)	1.33 (0.92, 1.93)
18-<35	1216 (87.2)	1053 (88.0)	163 (82.7)	Ref
≥35	27 (1.9)	20 (1.7)	7 (3.6)	**1.93 (1.01, 3.72)**
**Ethnic group**				
Hills (Pahadi)	101 (7.3)	89 (7.4)	12 (6.1)	Ref
Plains (Madeshi)	1292 (92.7)	1107 (92.6)	185 (93.9)	1.21 (0.70, 2.08)
**Height**				
Cm (mean ± SD)	150.8 ± 5.5	150.9 ± 5.6	150.1 ± 5.1	-0.80 (-1.63, 0.03)
**Weight**				
Kg (mean ± SD)	45.8 ± 7.0	46.0 ± 7.1	44.3 ± 5.8	**-1.67 (-2.72, -0.62)**
**BMI**				
Underweight (<18.5 kg)	418 (30.0)	353 (29.5)	65 (33.0)	1.09 (0.83, 1.44)
Normal weight (18.5-<25 kg)	893 (64.1)	766 (64.0)	127 (64.5)	Ref
Overweight or obese (≥25 kg)	83 (6.0)	78 (6.5)	5 (2.5)	0.42, (0.18, 1.01)
**High blood pressure**				
No	1384 (99.3)	1188 (99.2)	196 (99.5)	Ref
Yes	10 (0.7)	9 (0.8)	1 (0.5)	0.71 (0.11, 4.56)
**Gravidity**				
First pregnancy	395 (28.3)	332 (27.7)	63 (32.0)	1.23 (0.92, 1.63)
1-3 previous pregnancies	838 (60.1)	729 (60.9)	109 (55.3)	Ref
≥4 pregnancies	161 (11.5)	136 (11.4)	25 (12.7)	1.19 (0.80, 1.78)
**Urinary or vaginal infection+**				
No	1097 (78.7)	942 (78.7)	155 (78.7)	Ref
Yes	297 (21.3)	255 (21.3)	42 (21.3)	1.00 (0.73, 1.37)

For peer review only - http://bmjopen.bmj.com/site/about/guidelines.xhtml

**Literacy**				
No	751 (53.9)	638 (53.3)	113 (57.4)	Ref
Yes	643 (46.1)	559 (46.7)	84 (42.6)	0.87 (0.67, 1.13)
**Education (years)**				
0	751 (53.9)	634 (53.0)	117 (59.4)	Ref
1-9	386 (27.7)	343 (28.7)	43 (21.8)	**0.72 (0.52, 0.99)**
≥10	256 (18.4)	219 (18.3)	37 (18.8)	0.93 (0.66, 1.31)
**Electricity**				
No	114 (8.2)	97 (8.1)	17 (8.6)	Ref
Yes	1279 (91.8)	1099 (91.9)	180 (91.4)	0.94 (0.60, 1.49)
**House construction material**				
None, thatch, sticks, or bamboo	858 (61.6)	749 (62.6)	109 (55.3)	Ref
Wood planks, bricks, or stone	535 (38.4)	447 (37.4)	88 (44.7)	**1.29 (1.00, 1.68)**
**House roof material**				
None, plastic, thatch, or grass	110 (7.9)	92 (7.7)	18 (9.1)	Ref
Tile/tin/concrete	1283 (92.1)	1104 (92.3)	179 (90.9)	0.85 (0.55, 1.33)
**Latrine**				
No latrine	601 (43.1)	515 (43.1)	86 (43.7)	Ref
Brick, concrete, or pit latrine	792 (56.9)	681 (56.9)	111 (56.3)	0.98 (0.75, 1.27)

Data presented as No. (%) unless otherwise noted

* T-test or relative risk and 95% confidence interval as appropriate

+ Self-reported symptoms of painful urination or foul smelling vaginal discharge during pregnancy

For peer review only

For peer review only - http://bmjopen.bmj.com/site/about/guidelines.xhtml

According to our definition, 554 (39.7%) participants had gingival inflammation and 840 (60.3%) were healthy (Table 2). Of those with gingival inflammation, 445 (80.3%) had localized gingival inflammation (BOP $<30\%$) and 109 (19.7%) generalized gingival inflammation (BOP $\geq30\%$). Most participants (n=1,105, 79.3%) had at least one site with BOP. According to our secondary exposure definitions, 54 participants (3.9%) had mild and 4 (0.3%) moderate periodontitis. More participants (n=120, 8.6%) had ≥1 site with PD ≥4 mm. Mean CAL was equivalent to mean PD, with nearly all of CAL ≥4 mm due to pocketing secondary to gingival enlargement, indicating very little recession of the gingiva in this population.

For peer review only - http://bmjopen.bmj.com/site/about/guidelines.xhtml

Table 2: Periodontal disease status (n=1,394)

Characteristic	All	Term	Preterm	Rate of preterm
**Health and gingival inflammation**				
Health (All sites PD ≤3 mm and BOP <10%)	840 (60.3)	727 (60.7)	113 (57.4)	13.5%
Gingival inflammation (BOP ≥10% and/or PD ≥4 mm)	554 (39.7)	470 (39.3)	84 (42.6)	15.2%
- Localized gingival inflammation (BOP <30%)	445 (80.3)	379 (80.6)	66 (78.6)	14.8%
- Proportion with no sites PD ≥4 mm	354 (79.6)	299 (78.9)	55 (83.3)	15.5%
- Proportion with ≥1 sites PD ≥4 mm	91 (20.5)	80 (21.2)	11 (16.7)	12.1%
- Generalized gingival inflammation (BOP ≥30%)	109 (19.7)	91 (19.4)	18 (21.4)	16.5%
- Proportion with no sites PD ≥4 mm	80 (73.4)	66 (72.5)	14 (77.8)	17.5%
- Proportion with ≥1 sites PD ≥4 mm	29 (26.6)	25 (27.5)	4 (22.2)	13.8%
**Bleeding on probing (BOP)**				
Percent of sites BOP (mean ± SD)	10.2 ± 12.3	10.1 ± 12.4	10.6 ± 12.0	14.1%*
No sites BOP	289 (20.7)	249 (20.8)	40 (20.3)	13.8%
≥1 site BOP	1,105 (79.3)	949 (79.2)	157 (79.7)	14.2%
≥1 site BOP & BOP <10%	601 (43.1)	521 (43.5)	80 (40.6)	13.3%
BOP ≥10% & BOP <30%	395 (28.3)	336 (28.1)	59 (30.0)	14.9%
BOP ≥30%	109 (7.8)	91 (7.6)	18 (9.1)	16.5%
**Probing depth (PD)**				
Mean PD (mm) (mean ± SD)	1.7 ± 0.3	1.7 ± 0.3	1.7 ± 0.3	14.1%*
Mean PD at direct sites (mm) (mean ± SD)	1.5 ± 0.3	1.5 ± 0.3	1.5 ± 0.2	14.1%*
Percent of sites PD ≥4 mm (mean ± SD)	0.2 ± 1.0	0.2 ± 1.0	0.1 ± 0.9	14.1%*
≥1 site PD ≥4 mm	120 (8.6)	105 (8.8)	15 (7.6)	12.5%
**Clinical attachment loss (CAL)**				
Mean CAL at direct sites (mm) (mean ± SD)	1.7 ± 0.3	1.7 ± 0.3	1.7 ± 0.3	14.1%*
≥1 site recession ≥1 mm	173 (12.4)	154 (12.9)	19 (9.6)	11.0%
≥1 site CAL ≥4 mm	196 (14.1)	172 (14.4)	24 (12.2)	12.2%
Percent of CAL ≥4 mm due to pocketing (mean ± SD)	99.6 ± 1.5	99.6 ± 1.5	99.8 ± 0.9	14.1%*
Percent of CAL ≥4 mm due to recession (mean ± SD)	0.4 ± 1.5	0.4 ± 1.5	0.2 ± 0.9	14.1%*

Data presented as No. (%) unless otherwise noted

* Preterm birth rate among all participants

For peer review only - http://bmjopen.bmj.com/site/about/guidelines.xhtml

Several poor oral hygiene behaviors were associated with increased risk of preterm birth, including extended teeth cleaning time (≥30 mins vs. <5 mins: RR 2.48, 95% CI: 1.41, 4.39), use of a non-traditional dentifrice (i.e., sand, ash, oil, or gul) (RR 2.22, 95% CI: 1.44, 3.42). Teeth cleaning episodes per week (≥14 vs. <7 times: RR 0.55, 95% CI: 0.29, 1.02) was associated with decreased risk of preterm birth. Risk of preterm birth increased with a dose response pattern for women who practiced multiple poor oral hygiene behaviors, defined as extended cleaning time per episode (≥5 minutes), infrequent cleaning episodes (<7 times per week), and use of a traditional dentifrice. Incidence of preterm birth was 17/208 (8.2%) for women with no poor behaviors, 155/1,098 (14.1%) for women with one, and 25/88 (28.4%) for those with two.

Primary analysis: Association between maternal gingival inflammation and preterm birth

Incidence of preterm birth by periodontal status was 113/840 (13.5%) for health and 84/554 (15.2%) for gingival inflammation. Incidence of preterm birth increased from 120/890 (13.5%) for women with either health (no sites BOP) or BOP <10% to 59/395 (14.9%) for women with localized gingival inflammation (BOP ≥10% & <30%) to 18/109 (16.5%) for women with generalized gingival inflammation (BOP ≥30%).

Our binary definition of gingival inflammation (BOP ≥10%) was not associated with preterm birth in the crude model (RR 1.13, 95% CI: 0.87, 1.46) or after adjusting for confounders (aRR 1.16, 95% CI: 0.90, 1.51). In the final adjusted model, increasing severity of gingival inflammation was associated with a non-significant increase in risk of preterm birth (BOP ≥30% vs. no BOP: aRR 1.37, 95% CI: 0.81, 2.32) (Table 3). In this model, several oral hygiene behaviors were associated with preterm birth. Extended cleaning time (≥30 mins vs. <5 mins:

For peer review only

aRR 2.44, 95% CI: 1.34, 4.43), use of a traditional dentifrice (n=53, 3.9%) (aRR 2.17, 95% CI: 1.39, 3.38), and fewer teeth cleaning episodes per week (≥14 times vs. 1-6 times: aRR 0.46, 95% CI: 0.25, 0.84) were associated with increased risk of preterm birth.

For peer review only

For peer review only - http://bmjopen.bmj.com/site/about/guidelines.xhtml

Table 3: Adjusted relative risks (aRR) between maternal gingival inflammation and preterm birth

	Preterm birth aRR (95% CI)			
**Characteristic**	**Unadjusted model (n=1,394)**	**Model 1 (n=1,393)**	**Model 2 (n=1,358)**	**Final model (n=1,357)**
**Gingival inflammation**				
No sites BOP	Ref	Ref	Ref	Ref
≥1 site BOP & BOP <10%	0.96 (0.68, 1.37)	0.99 (0.70, 1.40)	1.08 (0.76, 1.54)	1.07 (0.75, 1.51)
BOP ≥10% & BOP <30%	1.08 (0.74, 1.57)	1.11 (0.77, 1.61)	1.19 (0.82, 1.73)	1.17 (0.80, 1.69)
BOP ≥30%	1.19 (0.72, 1.99)	1.24 (0.74, 2.08)	1.34 (0.79, 2.28)	1.37 (0.81, 2.32)
**Age (years)**				
<18		1.21 (0.80, 1.85)	1.17 (0.77, 1.76)	1.21 (0.80, 1.84)
18-<35		Ref	Ref	Ref
≥35		**2.17 (1.14, 4.11)**	1.82 (0.95, 3.49)	1.65 (0.88, 3.10)
**Pahadi/Madeshi**				
Pahadi		Ref	Ref	Ref
Madeshi		1.13 (0.65, 1.97)	0.95 (0.55, 1.66)	0.97 (0.55, 1.70)
**BMI (kg)**				
Underweight (<18.5)		1.10 (0.83, 1.45)	1.11 (0.85, 1.47)	1.15 (0.87, 1.52)
Normal weight (18.5-<25)		Ref	Ref	Ref
Overweight or obese (≥25)		0.45 (0.18, 1.08)	0.49 (0.20, 1.18)	0.47 (0.20, 1.13)
**Primiparous**				
No		Ref	Ref	Ref
Yes		1.17 (0.86, 1.60)	1.25 (0.92, 1.71)	1.26 (0.91, 1.75)
**Multiple births**				
No		Ref	Ref	Ref
Yes		**4.46 (2.88, 6.92)**	**3.76 (2.46, 5.75)**	**3.38 (2.09, 5.46)**
**Teeth cleaning time per episode (mins)**				
<5			Ref	Ref
5-<30			**1.77 (1.07, 2.94)**	**1.83 (1.11, 3.04)**
≥30			**2.37 (1.31, 4.27)**	**2.44 (1.34, 4.43)**
**Teeth cleaning episodes per week**				
1-6			Ref	Ref

For peer review only - http://bmjopen.bmj.com/site/about/guidelines.xhtml

For peer review only

7-13	0.64 (0.39, 1.05)	**0.62 (0.39, 1.00)**
≥14	**0.48 (0.26, 0.89)**	**0.46 (0.25, 0.84)**
**Use of traditional dentifrice**		
No	Ref	Ref
Yes	**2.13 (1.37, 3.31)**	**2.17 (1.39, 3.38)**
**Literacy**		
No		Ref
Yes		1.51 (0.85, 2.66)
**Education (years)**		
0		Ref
1-9		**0.50 (0.27, 0.92)**
≥10		0.65 (0.35, 1.21)
**House construction material**		
None, plastic, thatch, or grass		Ref
Wood planks, bricks, or stone		**1.47 (1.11, 1.93)**
**Roof construction material**		
None, plastic, thatch, or grass		Ref
Tin, tile, or concrete		0.85 (0.55, 1.33)

For peer review only

For peer review only - http://bmjopen.bmj.com/site/about/guidelines.xhtml

Secondary analysis: Stratification by gestational age at periodontal examination

In a secondary analysis, participants were stratified by their trimester at the time of the periodontal examination (1st trimester: n=574; 2nd trimester: n=820). Among women with dental exams in the first trimester, incidence of preterm birth increased from 47/381 (12.3%) for women with either health (no sites BOP) or BOP <10% to 28/158 (17.7%) for women with localized gingival inflammation (BOP ≥10% & <30%) to 8/35 (22.9%) for women with generalized gingival inflammation (BOP ≥30%). Our binary definition of gingival inflammation (BOP ≥10%) in the first trimester was associated with preterm (aRR 1.62, 95% CI: 1.08, 2.42), but not in the second trimester (aRR 0.92, 95% CI: 0.65, 1.30), after adjusting for confounders. In the final adjusted model stratified by trimester, there was a positive relationship between gingival inflammation and risk of preterm birth among women in the first trimester (BOP ≥30% vs. no BOP: aRR 2.57, 95% CI: 1.11, 5.95), but not among women in their second trimester (BOP ≥30% vs. no BOP: aRR 1.05, 95% CI: 0.52, 2.11) (Table 4).

Our secondary exposure definition for mild periodontitis was not associated with preterm in a binary comparison or the crude or adjusted regression models for our primary or secondary analyses. These analyses were not conducted for moderate periodontitis given the low prevalence of the condition in this study population.

For peer review only - http://bmjopen.bmj.com/site/about/guidelines.xhtml

Table 4: Adjusted relative risks (aRR) between maternal gingival inflammation and preterm birth stratified by trimester at periodontal examination visit

	Preterm birth aRR (95% CI)	
**Characteristic**	**Women in 1st trimester (n=559)**	**Women in 2nd trimester (n=798)**
**Gingival inflammation**		
No sites BOP	Ref	Ref
≥1 site BOP & BOP <10%	1.51 (0.81, 2.84)	0.93 (0.60, 1.44)
BOP ≥10% & BOP <30%	**1.91 (1.00, 3.63)**	0.88 (0.55, 1.42)
BOP ≥30%	**2.57 (1.11, 5.95)**	1.05 (0.52, 2.11)
**Age (years)**		
<18	1.06 (0.52, 2.18)	1.42 (0.85, 2.36)
18-<35	Ref	Ref
≥35	0.97 (0.28, 3.38)	**2.42 (1.14, 5.14)**
**Pahadi/Madeshi**		
Pahadi	Ref	Ref
Madeshi	1.15 (0.46, 2.90)	0.82 (0.42, 1.60)
**BMI (kg)**		
Underweight (<18.5)	1.44 (0.95, 2.18)	0.96 (0.65, 1.42)
Normal weight (18.5-<25)	Ref	Ref
Overweight or obese (≥25)	0.72 (0.19, 2.78)	0.33 (0.11, 1.06)
**Primiparous**		
No	Ref	Ref
Yes	1.41 (0.84, 2.34)	1.13 (0.74, 1.72)
**Multiple births**		
No	Ref	Ref
Yes	2.57 (0.92, 7.15)	**4.38 (2.57, 7.47)**
**Teeth cleaning time per episode (mins)**		
<5	Ref	Ref
5-<30	1.40 (0.71, 2.75)	**2.42 (1.13, 5.19)**
≥30	2.06 (0.92, 4.57)	**2.93 (1.19, 7.23)**
**Teeth cleaning episodes per week**		
1-6	Ref	Ref
7-13	0.87 (0.39, 1.93)	**0.49 (0.28, 0.85)**
≥14	0.49 (0.18, 1.31)	**0.46 (0.22, 0.95)**
**Use of traditional dentifrice**		
No	Ref	Ref
Yes	1.61 (0.63, 4.10)	**2.58 (1.56, 4.26)**
**Literacy**		
No	Ref	Ref
Yes	1.97 (0.78, 4.96)	1.25 (0.60, 2.61)
**Education (years)**		
0	Ref	Ref
1-9	**0.34 (0.13, 0.89)**	0.67 (0.31, 1.45)

For peer review only

≥10	0.41 (0.15, 1.11)	0.93 (0.41, 2.10)
**House construction material**		
None, plastic, thatch, or grass	Ref	Ref
Wood planks, bricks, or stone	1.33 (0.84, 2.11)	**1.57 (1.09, 2.25)**
**Roof construction material**		
None, plastic, thatch, or grass	Ref	Ref
Tin, tile, or concrete	0.80 (0.39, 1.65)	0.90 (0.51, 1.59)

For peer review only

DISCUSSION

In our community-based, prospective cohort study, gingival inflammation was an independent risk factor for preterm birth among women examined during their first, but not second, trimester, after adjusting for risk factors. Our finding suggests that gingival infection and inflammatory burden early in pregnancy, potentially originating prior to conception, are responsible for the observed relationship, which becomes obscured in the second trimester by the presence of women with initially healthy periodontal conditions who develop pregnancy-induced gingival inflammation. This result is consistent with studies that have reported that active, and especially progressive, periodontal infection is most harmful early in pregnancy.[13] This also supports the hypothesis, proposed to explain the mixed results of RCTs of this association, that intervening on women with periodontal disease later in pregnancy, or even after conception, may be too late to affect the proposed causal pathway.[32]

Most previous studies have considered the relationship between varying definitions and severities of periodontitis and preterm birth. A meta-analysis of seven cohort studies estimated a pooled relative risk of preterm of 1.70 (1.03, 2.81) for pregnant women with periodontitis versus health.[10] Prevalence of periodontitis in our study population was low, although not dissimilar to previous surveys of periodontal conditions among this age group in Nepal.[33] Additional data on the clinical periodontal characteristics and oral hygiene behaviors of women in our study are published in detail elsewhere.[31] The low prevalence of periodontitis in this population is likely attributable to the population's low age (median 22.2, IQR: 19.7, 25.5; mean 23.0, SD: 4.6) and absence of other common risk factors for the condition. An unpublished review of community-based studies of the periodontal disease and preterm birth relationship in low- and middle-income countries conducted by our study team found mean maternal ages ranging from 26 to 29.

For peer review only - http://bmjopen.bmj.com/site/about/guidelines.xhtml

Unlike the majority of studies of this association, particularly in high-income countries, the prevalence of important confounders, such as smoking, alcohol use, and morbidities associated with chronic disease, were nearly absent in our study population. An association between periodontitis and preterm birth may have been seen in our population had there been a higher prevalence and severity of periodontitis, as would be expected in an older population.

Few observational or interventional studies have considered gingival inflammation as the primary exposure definition when investigating the relationship between periodontal disease and preterm birth. A cohort study by Kruse et al. (2008) found an association between gingivitis and high risk of preterm birth among women without periodontitis in a hospital setting in Germany.[34] A trial by Lopez et al. (2005) of women with gingivitis in a hospital setting in Santiago, Chile, demonstrated a significantly higher risk of preterm low birth weight among women with gingivitis who received periodontal treatment after delivery compared to those that received treatment during pregnancy (<28 weeks) (aOR: 2.76, 95% CI: 1.29, 5.88).[35] Gingival inflammation requires less intensive treatment than periodontitis, and can include improved oral hygiene, use of an antiseptic oral rinse, and periodontal therapy, interventions that could be feasibly delivered in a rural community setting. While antiseptic oral rinses are known for their ability to reduce plaque and control inflammation, their action on pregnancy outcomes is underexplored.[36, 37] However, Jeffcoat et al. (2011) found a reduction in preterm birth in a high-risk population with the use of cetylpyridinium chloride oral rinse intervention.[38]

Severity of gingival inflammation, independent of traditional clinical measures of periodontitis, such as CAL or PD, has been associated with magnitude of bacteremia.[39] Transient

For peer review only - http://bmjopen.bmj.com/site/about/guidelines.xhtml

bacteremia, facilitated by ulceration, inflammation, and the increased vascular permeability of the gingiva, could pose increased risk for adverse pregnancy outcomes.[40] In this case, treatment of traditional clinical measures of periodontitis – without reducing infection, inflammation, and, potentially, the presence of specific harmful pathogens – could fail to disrupt the proposed causal pathway between exposure and outcome. Further, if such bacteremia were to occur early in pregnancy, treatment of clinical conditions later in pregnancy would be too late to eliminate exposure of the placenta and/or fetus to these pathogens, and hence would not decrease of risk of adverse pregnancy outcomes.[40]

Poor oral hygiene behaviors, including extended teeth cleaning time (≥5 mins), infrequent teeth cleaning (<7 times per week), and use of traditional dentifrice (i.e., sand, ash, oil, or gul) or datiwan, were also associated with increased risk of preterm birth in our analysis. Studies have shown that the mechanical manipulation involved in brushing, particularly frequent or forceful brushing or use of brushes with hard filaments, can lead to gingival abrasion, recession, and bacteremia.[41-43] Some have posited that bacteremia caused by mechanical manipulation of the gingiva involved in periodontal therapy may increase risk of adverse pregnancy outcomes, attenuating observation of any true effect in interventional studies of this association.[32] More research is needed to understand the extent to which poor oral hygiene behaviors in pregnant women, including common traditional practices, such as datiwan use, could – via local inflammatory responses or increased access of periodontal bacteria or their byproducts to the bloodstream – initiate a cascade of effects that ultimately influences the pregnancy outcome.

For peer review only - http://bmjopen.bmj.com/site/about/guidelines.xhtml

An important strength of our study was that we utilized a population-based sample, compared to previous observational studies of this relationship, which were primarily conducted in health facility settings, introducing risk of selection bias associated with the likelihood of women in the target population delivering in a particular facility. The potential for selection bias to influence the measure of association in a facility-based study is especially high in populations where a substantial proportion of women deliver at home, as is the case in this area of rural Nepal, where roughly half of women deliver in a facility. In unpublished review conducted by our study team, we found one study of this association, by Mobeen et al. (2008), that used a population-based sample.[44] Conducted in a periurban area of Hyderabad, Pakistan, and the authors reported significant associations of varying degrees between measures of periodontal disease and neonatal death, perinatal death, and stillbirth.[44]

A limitation of this study was the collection of clinical recession measures from only the direct buccal and lingual surfaces. In the absence of these data, we likely underestimated the burden of periodontal disease among pregnant women in this study, potentially attenuating our measure of association. We were unable to control for some confounders of this relationship, including previous preterm birth, certain chronic diseases (e.g., diabetes), and used only a proxy (self-reported symptoms) for urinary tract and vaginal infections. Conducting periodontal exams in participant homes, where light and other conditions are variable, may have introduced a level of measurement error in our exposure that could not be quantified or controlled for given the setting and logistical constraints of our study. Lastly, preterm birth was based upon maternal self-report of last menstrual period (LMP) instead of ultrasound examination. If our LMP estimates were less accurate among women examined later in pregnancy, due to longer maternal recall relative

For peer review only - http://bmjopen.bmj.com/site/about/guidelines.xhtml

For peer review only

to women examined earlier in pregnancy, this could have resulted in non-differential misclassification of our binary outcome, preterm birth, which would tend to attenuate observation of a true relationship among women examined in the second trimester.

CONCLUSION

Our study identified gingival inflammation as an independent risk factor for preterm birth among women examined early in pregnancy. Women non-compliant with oral hygiene care recommendations were at substantially increased risk of preterm birth, highlighting the importance of adherence to proper home care in pregnant women and women expecting to become pregnant. Future studies should evaluate the effectiveness of community-based oral health interventions that specifically target gingival inflammation– delivered to women early in or prior to pregnancy – on the incidence of preterm birth and other adverse pregnancy outcomes in low-income countries with high risk for these outcomes.

For peer review only - http://bmjopen.bmj.com/site/about/guidelines.xhtml

Acknowledgements: None.

Competing interests: The authors declare that they have no competing interests.

Funding source: This work was supported by the National Institute for Child Health and Development [HD060712] and the Bill & Melinda Gates Foundation [OPP1084399, OPP1131701].

Trial registration: ClinicalTrials.gov Identifier: NCT01177111 (Nepal Oil Massage Study) and NCT02788786 (Pilot Trial).

Patients or public involvement: Patients or the public were not involved in the design, or conduct, or reporting, or dissemination plans of our research.

Data availability: All data files, codebooks, and related manuscripts are available from the JHU Data Archive (https://doi.org/10.7281/T1/ZPGBJW).

Abbreviations:

Adjusted relative risk (aRR)

Bleeding on probing (BOP)

Body mass index (BMI)

Cemento-enamel junction (CEJ)

Clinical attachment loss (CAL)

Confidence intervals (CI)

Gingival margin (GM)

Nepal Oil Massage Study (NOMS)

Relative risk (RR)

Probing depth (PD)

Standard deviation (SD)

Authors' contribution:

For peer review only

Dr. Erchick conceptualized and designed the study, developed field implementation protocols, led data collection in the field, conducted the analysis, and wrote the manuscript.

Dr. Khatry conceptualized and designed the study, oversaw field implementation and ensured quality data collection, and provided comments on the manuscript.

Dr. Agrawal conceptualized and designed the study, trained and oversaw the data collectors, and provided comments on the manuscript.

Dr. Katz conceptualized and designed the study, ensured quality data collection, advised on analytic approach, and provided comments on the manuscript.

Mr. LeClerq conceptualized and designed the study, supported overall implementation in the field, and provided comments on the manuscript.

Mr. Rai contributed to field implementation, ensured quality control of field procedures, and provided comments on the manuscript.

Dr. Reynolds conceptualized and designed the study, contributed to the analysis and interpretation of the results, and provided comments on the manuscript.

Dr. Mullany conceptualized and designed the study, obtained funding for the study, oversaw implementation of data collection, obtained ethical approvals, advised on analytic approach, and provided edits and comments on the manuscript.

All authors approved the final manuscript as submitted and agree to be accountable for all aspects of the work.

For peer review only - http://bmjopen.bmj.com/site/about/guidelines.xhtml

REFERENCES

1. United Nations Inter-agency Group for Child Mortality Estimation (UN IGME). Levels & Trends in Child Mortality. In: UNICEF, ed. New York, 2018.

2. Blencowe H, Cousens S, Chou D, et al. Born too soon: the global epidemiology of 15 million preterm births. *Reproductive health* 2013;10 Suppl 1:S2. doi: 10.1186/1742-4755-10-s1-s2 [published Online First: 2014/03/15]

3. Barros FC, Bhutta ZA, Batra M, et al. Global report on preterm birth and stillbirth (3 of 7): evidence for effectiveness of interventions. *BMC pregnancy and childbirth* 2010;10 Suppl 1:S3. doi: 10.1186/1471-2393-10-s1-s3 [published Online First: 2010/03/27]

4. Brown LJ, Loe H. Prevalence, extent, severity and progression of periodontal disease. *Periodontology 2000* 1993;2:57-71. [published Online First: 1993/06/01]

5. Loe H, Silness J. Periodontal disease in pregnancy. I. Prevalence and severity. *Acta odontologica Scandinavica* 1963;21:533-51. [published Online First: 1963/12/01]

6. Laine MA. Effect of pregnancy on periodontal and dental health. *Acta odontologica Scandinavica* 2002;605:257-64. [published Online First: 2002/11/07]

7. Figuero E, Carrillo-de-Albornoz A, Martin C, et al. Effect of pregnancy on gingival inflammation in systemically healthy women: a systematic review. *Journal of clinical periodontology* 2013;405:457-73. doi: 10.1111/jcpe.12053 [published Online First: 2013/04/06]

8. Wu M, Chen SW, Jiang SY. Relationship between gingival inflammation and pregnancy. *Mediators of inflammation* 2015;2015:623427. doi: 10.1155/2015/623427 [published Online First: 2015/04/16]

9. Offenbacher S, Katz V, Fertik G, et al. Periodontal infection as a possible risk factor for preterm low birth weight. *Journal of periodontology* 1996;6710 Suppl:1103-13. doi: 10.1902/jop.1996.67.10s.1103 [published Online First: 1996/10/01]

10. Chambrone L, Guglielmetti MR, Pannuti CM, et al. Evidence grade associating periodontitis to preterm birth and/or low birth weight: I. A systematic review of prospective cohort studies. *Journal of clinical periodontology* 2011;389:795-808. doi: 10.1111/j.1600-051X.2011.01755.x [published Online First: 2011/06/29]

11. Ide M, Papapanou PN. Epidemiology of association between maternal periodontal disease and adverse pregnancy outcomes--systematic review. *Journal of periodontology* 2013;844 Suppl:S181-94. doi: 10.1902/jop.2013.134009 [published Online First: 2013/05/03]

For peer review only - http://bmjopen.bmj.com/site/about/guidelines.xhtml

12. Offenbacher S, Lieff S, Boggess KA, et al. Maternal periodontitis and prematurity. Part I: Obstetric outcome of prematurity and growth restriction. *Annals of periodontology / the American Academy of Periodontology* 2001;61:164-74. doi: 10.1902/annals.2001.6.1.164 [published Online First: 2002/03/13]

13. Offenbacher S, Boggess KA, Murtha AP, et al. Progressive periodontal disease and risk of very preterm delivery. *Obstetrics and gynecology* 2006;1071:29-36. doi: 10.1097/01.aog.0000190212.87012.96 [published Online First: 2006/01/06]

14. Jeffcoat MK, Geurs NC, Reddy MS, et al. Periodontal infection and preterm birth: results of a prospective study. *Journal of the American Dental Association (1939)* 2001;1327:875-80. [published Online First: 2001/08/02]

15. Michalowicz BS, Hodges JS, DiAngelis AJ, et al. Treatment of periodontal disease and the risk of preterm birth. *The New England journal of medicine* 2006;35518:1885-94. doi: 10.1056/NEJMoa062249 [published Online First: 2006/11/03]

16. Offenbacher S, Beck JD, Jared HL, et al. Effects of periodontal therapy on rate of preterm delivery: a randomized controlled trial. *Obstetrics and gynecology* 2009;1143:551-9. doi: 10.1097/AOG.0b013e3181b1341f [published Online First: 2009/08/25]

17. Offenbacher S, Lin D, Strauss R, et al. Effects of periodontal therapy during pregnancy on periodontal status, biologic parameters, and pregnancy outcomes: a pilot study. *Journal of periodontology* 2006;7712:2011-24. doi: 10.1902/jop.2006.060047 [published Online First: 2007/01/11]

18. Iheozor-Ejiofor Z, Middleton P, Esposito M, et al. Treating periodontal disease for preventing adverse birth outcomes in pregnant women. *The Cochrane database of systematic reviews* 2017;6:Cd005297. doi: 10.1002/14651858.CD005297.pub3 [published Online First: 2017/06/13]

19. Kim AJ, Lo AJ, Pullin DA, et al. Scaling and root planing treatment for periodontitis to reduce preterm birth and low birth weight: a systematic review and meta-analysis of randomized controlled trials. *Journal of periodontology* 2012;8312:1508-19. doi: 10.1902/jop.2012.110636 [published Online First: 2012/03/02]

20. Cetin I, Pileri P, Villa A, et al. Pathogenic mechanisms linking periodontal diseases with adverse pregnancy outcomes. *Reproductive sciences (Thousand Oaks, Calif)* 2012;196:633-41. doi: 10.1177/1933719111432871 [published Online First: 2012/03/17]

For peer review only

For peer review only - http://bmjopen.bmj.com/site/about/guidelines.xhtml

21. Jeffcoat MK, Jeffcoat RL, Tanna N, et al. Association of a common genetic factor, PTGER3, with outcome of periodontal therapy and preterm birth. *Journal of periodontology* 2014;853:446-54. doi: 10.1902/jop.2013.130006 [published Online First: 2013/06/29]

22. Armitage GC. Development of a classification system for periodontal diseases and conditions. *Annals of periodontology / the American Academy of Periodontology* 1999;41:1-6. doi: 10.1902/annals.1999.4.1.1 [published Online First: 2000/06/23]

23. Chapple ILC, Mealey BL, Van Dyke TE, et al. Periodontal health and gingival diseases and conditions on an intact and a reduced periodontium: Consensus report of workgroup 1 of the 2017 World Workshop on the Classification of Periodontal and Peri-Implant Diseases and Conditions. *Journal of periodontology* 2018;89 Suppl 1:S74-s84. doi: 10.1002/jper.17-0719 [published Online First: 2018/06/22]

24. Trombelli L, Farina R, Silva CO, et al. Plaque-induced gingivitis: Case definition and diagnostic considerations. *Journal of periodontology* 2018;89 Suppl 1:S46-s73. doi: 10.1002/jper.17-0576 [published Online First: 2018/06/22]

25. Holtfreter B, Albandar JM, Dietrich T, et al. Standards for reporting chronic periodontitis prevalence and severity in epidemiologic studies: Proposed standards from the Joint EU/USA Periodontal Epidemiology Working Group. *Journal of clinical periodontology* 2015;425:407-12. doi: 10.1111/jcpe.12392 [published Online First: 2015/03/27]

26. Papapanou PN, Sanz M, Buduneli N, et al. Periodontitis: Consensus report of workgroup 2 of the 2017 World Workshop on the Classification of Periodontal and Peri-Implant Diseases and Conditions. *Journal of periodontology* 2018;89 Suppl 1:S173-s82. doi: 10.1002/jper.17-0721 [published Online First: 2018/06/22]

27. Tonetti MS, Greenwell H, Kornman KS. Staging and grading of periodontitis: Framework and proposal of a new classification and case definition. *Journal of periodontology* 2018;89 Suppl 1:S159-s72. doi: 10.1002/jper.18-0006 [published Online First: 2018/06/22]

28. World Health Organization. Epidemiology, etiology, and prevention of periodontal diseases. *World Health Organization technical report series* 1978 [published Online First: 1978/01/01]

29. Eke PI, Page RC, Wei L, et al. Update of the case definitions for population-based surveillance of periodontitis. *Journal of periodontology* 2012;8312:1449-54. doi: 10.1902/jop.2012.110664 [published Online First: 2012/03/17]

For peer review only

30. Page RC, Eke PI. Case definitions for use in population-based surveillance of periodontitis. *Journal of periodontology* 2007;787 Suppl:1387-99. doi: 10.1902/jop.2007.060264 [published Online First: 2007/08/19]
31. Erchick DJ, Rai B, Agrawal NK, et al. Oral hygiene, prevalence of gingivitis, and associated risk factors among pregnant women in Sarlahi District, Nepal. *BMC oral health* 2019;191:2. doi: 10.1186/s12903-018-0681-5 [published Online First: 2019/01/07]
32. Xiong X, Buekens P, Goldenberg RL, et al. Optimal timing of periodontal disease treatment for prevention of adverse pregnancy outcomes: before or during pregnancy? *American journal of obstetrics and gynecology* 2011;2052:111.e1-6. doi: 10.1016/j.ajog.2011.03.017 [published Online First: 2011/05/31]
33. Yee R, Mishra P. Nepal National Oral Health 'Pathfinder' Survey 2004. Kathmandu, Nepal: Oral Health Focal Point, Ministry of Health, 2004.
34. Kruse AB, Kuerschner AC, Kunze M, et al. Association between high risk for preterm birth and changes in gingiva parameters during pregnancy-a prospective cohort study. *Clinical oral investigations* 2018;223:1263-71. doi: 10.1007/s00784-017-2209-9 [published Online First: 2017/10/04]
35. Lopez NJ, Da Silva I, Ipinza J, et al. Periodontal therapy reduces the rate of preterm low birth weight in women with pregnancy-associated gingivitis. *Journal of periodontology* 2005;7611 Suppl:2144-53. doi: 10.1902/jop.2005.76.11-S.2144 [published Online First: 2005/11/10]
36. Sharma S, Saimbi CS, Koirala B, et al. Effect of various mouthwashes on the levels of interleukin-2 and interferon-gamma in chronic gingivitis. *The Journal of clinical pediatric dentistry* 2008;322:111-4. [published Online First: 2008/04/09]
37. Santos A. Evidence-based control of plaque and gingivitis. *Journal of clinical periodontology* 2003;30 Suppl 5:13-6. [published Online First: 2003/06/06]
38. Jeffcoat M, Parry S, Gerlach RW, et al. Use of alcohol-free antimicrobial mouth rinse is associated with decreased incidence of preterm birth in a high-risk population. *American journal of obstetrics and gynecology* 2011;2054:382.e1-6. doi: 10.1016/j.ajog.2011.07.016 [published Online First: 2011/11/16]
39. Forner L, Larsen T, Kilian M, et al. Incidence of bacteremia after chewing, tooth brushing and scaling in individuals with periodontal inflammation. *Journal of clinical periodontology* 2006;336:401-7. doi: 10.1111/j.1600-051X.2006.00924.x [published Online First: 2006/05/09]

For peer review only

For peer review only - http://bmjopen.bmj.com/site/about/guidelines.xhtml

40. Madianos PN, Bobetsis YA, Offenbacher S. Adverse pregnancy outcomes (APOs) and periodontal disease: pathogenic mechanisms. *Journal of periodontology* 2013;844 Suppl:S170-80. doi: 10.1902/jop.2013.1340015 [published Online First: 2013/05/03]
41. Litonjua LA, Andreana S, Bush PJ, et al. Toothbrushing and gingival recession. *International dental journal* 2003;532:67-72. [published Online First: 2003/05/07]
42. Khocht A, Simon G, Person P, et al. Gingival recession in relation to history of hard toothbrush use. *Journal of periodontology* 1993;649:900-5. doi: 10.1902/jop.1993.64.9.900 [published Online First: 1993/09/01]
43. Sconyers JR, Crawford JJ, Moriarty JD. Relationship of bacteremia to toothbrushing in patients with periodontitis. *Journal of the American Dental Association (1939)* 1973;873:616-22. doi: 10.14219/jada.archive.1973.0453 [published Online First: 1973/09/01]
44. Mobeen N, Jehan I, Banday N, et al. Periodontal disease and adverse birth outcomes: a study from Pakistan. *American journal of obstetrics and gynecology* 2008;1985:514.e1-8. doi: 10.1016/j.ajog.2008.03.010 [published Online First: 2008/05/06]

For peer review only

Figure 1: Cohort study participation flow chart

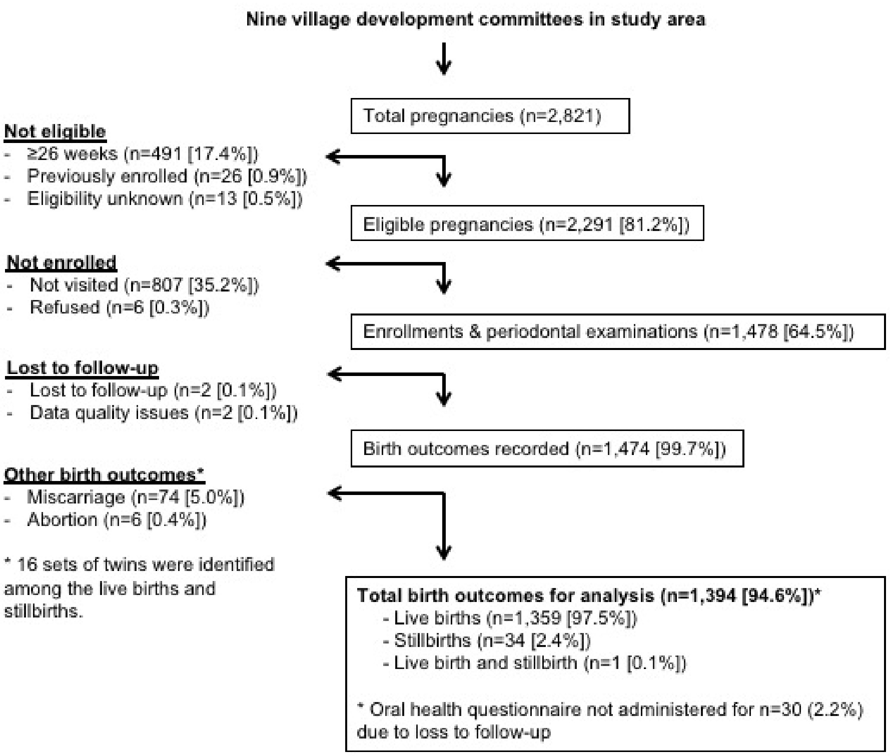

w only

STROBE Statement—Checklist of items that should be included in reports of ***cohort studies***

	Item No	Recommendation	Page No
**Title and abstract**	1	(*a*) Indicate the study's design with a commonly used term in the title or the abstract<br>(*b*) Provide in the abstract an informative and balanced summary of what was done and what was found	1-3
**Introduction**			
Background/rationale	2	Explain the scientific background and rationale for the investigation being reported	4-5
Objectives	3	State specific objectives, including any prespecified hypotheses	5
**Methods**			
Study design	4	Present key elements of study design early in the paper	6
Setting	5	Describe the setting, locations, and relevant dates, including periods of recruitment, exposure, follow-up, and data collection	6-7
Participants	6	(*a*) Give the eligibility criteria, and the sources and methods of selection of participants. Describe methods of follow-up<br>(*b*) For matched studies, give matching criteria and number of exposed and unexposed	6-9
Variables	7	Clearly define all outcomes, exposures, predictors, potential confounders, and effect modifiers. Give diagnostic criteria, if applicable	7-9
Data sources/ measurement	8*	For each variable of interest, give sources of data and details of methods of assessment (measurement). Describe comparability of assessment methods if there is more than one group	7-9
Bias	9	Describe any efforts to address potential sources of bias	8-9
Study size	10	Explain how the study size was arrived at	8
Quantitative variables	11	Explain how quantitative variables were handled in the analyses. If applicable, describe which groupings were chosen and why	8-9
Statistical methods	12	(*a*) Describe all statistical methods, including those used to control for confounding<br>(*b*) Describe any methods used to examine subgroups and interactions<br>(*c*) Explain how missing data were addressed<br>(*d*) If applicable, explain how loss to follow-up was addressed<br>(*e*) Describe any sensitivity analyses	8-9
**Results**			
Participants	13*	(a) Report numbers of individuals at each stage of study—eg numbers potentially eligible, examined for eligibility, confirmed eligible, included in the study, completing follow-up, and analysed<br>(b) Give reasons for non-participation at each stage<br>(c) Consider use of a flow diagram	10
Descriptive data	14*	(a) Give characteristics of study participants (eg demographic, clinical, social) and information on exposures and potential confounders<br>(b) Indicate number of participants with missing data for each variable of interest<br>(c) Summarise follow-up time (eg, average and total amount)	10-11
Outcome data	15*	Report numbers of outcome events or summary measures over time	10

For peer review only

For peer review only - http://bmjopen.bmj.com/site/about/guidelines.xhtml

Main results	16	(*a*) Give unadjusted estimates and, if applicable, confounder-adjusted estimates and their precision (eg, 95% confidence interval). Make clear which confounders were adjusted for and why they were included<br>(*b*) Report category boundaries when continuous variables were categorized<br>(*c*) If relevant, consider translating estimates of relative risk into absolute risk for a meaningful time period	15-19
Other analyses	17	Report other analyses done—eg analyses of subgroups and interactions, and sensitivity analyses	20-22
**Discussion**			
Key results	18	Summarise key results with reference to study objectives	23
Limitations	19	Discuss limitations of the study, taking into account sources of potential bias or imprecision. Discuss both direction and magnitude of any potential bias	26-27
Interpretation	20	Give a cautious overall interpretation of results considering objectives, limitations, multiplicity of analyses, results from similar studies, and other relevant evidence	27
Generalisability	21	Discuss the generalisability (external validity) of the study results	27
**Other information**			
Funding	22	Give the source of funding and the role of the funders for the present study and, if applicable, for the original study on which the present article is based	28

*Give information separately for exposed and unexposed groups.

Note: An Explanation and Elaboration article discusses each checklist item and gives methodological background and published examples of transparent reporting. The STROBE checklist is best used in conjunction with this article (freely available on the Web sites of PLoS Medicine at http://www.plosmedicine.org/, Annals of Internal Medicine at http://www.annals.org/, and Epidemiology at http://www.epidem.com/). Information on the STROBE Initiative is available at http://www.strobe-statement.org.

For peer review only

BMJ Open

Risk of preterm birth associated with maternal gingival inflammation and oral hygiene behaviors in rural Nepal

Journal:	*BMJ Open*
Manuscript ID	bmjopen-2019-036515.R1
Article Type:	Original research
Date Submitted by the Author:	05-May-2020
Complete List of Authors:	Erchick, Daniel; Johns Hopkins University Bloomberg School of Public Health, International Health<br>Khatry, Subarna; Nepal Nutrition Intervention Project<br>Agrawal, Nitin; Institute of Medicine, Tribhuhvan University, Department of Dentistry<br>Katz, Joanne; Johns Hopkins University Bloomberg School of Public Health, International Health<br>LeClerq, Steven; Johns Hopkins University Bloomberg School of Public Health, International Health; Nepal Nutrition Intervention Project Sarlahi<br>Rai, Bhola; Nepal Nutrition Intervention Project Sarlahi<br>Reynolds, Mark; University of Maryland School of Dentistry, Department of Advanced Oral Sciences and Therapeutics<br>Mullany, Luke C.; Johns Hopkins University Bloomberg School of Public Health, International Health
**Primary Subject Heading**:	Global health
Secondary Subject Heading:	Epidemiology, Dentistry and oral medicine, Obstetrics and gynaecology, Public health
Keywords:	EPIDEMIOLOGY, OBSTETRICS, ORAL MEDICINE, Community child health < PAEDIATRICS, PERINATOLOGY, PUBLIC HEALTH

SCHOLARONE™
Manuscripts

I, the Submitting Author has the right to grant and does grant on behalf of all authors of the Work (as defined in the below author licence), an exclusive licence and/or a non-exclusive licence for contributions from authors who are: i) UK Crown employees; ii) where BMJ has agreed a CC-BY licence shall apply, and/or iii) in accordance with the terms applicable for US Federal Government officers or employees acting as part of their official duties; on a worldwide, perpetual, irrevocable, royalty-free basis to BMJ Publishing Group Ltd ("BMJ") its licensees and where the relevant Journal is co-owned by BMJ to the co-owners of the Journal, to publish the Work in this journal and any other BMJ products and to exploit all rights, as set out in our licence.

The Submitting Author accepts and understands that any supply made under these terms is made by BMJ to the Submitting Author unless you are acting as an employee on behalf of your employer or a postgraduate student of an affiliated institution which is paying any applicable article publishing charge ("APC") for Open Access articles. Where the Submitting Author wishes to make the Work available on an Open Access basis (and intends to pay the relevant APC), the terms of reuse of such Open Access shall be governed by a Creative Commons licence – details of these licences and which Creative Commons licence will apply to this Work are set out in our licence referred to above.

Other than as permitted in any relevant BMJ Author's Self Archiving Policies, I confirm this Work has not been accepted for publication elsewhere, is not being considered for publication elsewhere and does not duplicate material already published. I confirm all authors consent to publication of this Work and authorise the granting of this licence.

For peer review only

Risk of preterm birth associated with maternal gingival inflammation and oral hygiene behaviors in rural Nepal

Daniel J. Erchick[a], PhD, MPH, Subarna K. Khatry[b], MBBS, Nitin K. Agrawal[c], MDS, MSc, Joanne Katz[a], ScD, MS, Steven C. LeClerq[a,b], MPH, Bhola Rai[b], Mark A. Reynolds[d], DDS, PhD, MA, Luke C. Mullany[a], PhD, MHS, MS

Affiliations:

[a]Department of International Health, Johns Hopkins Bloomberg School of Public Health, Baltimore, MD, United States

[b]Nepal Nutrition Intervention Project – Sarlahi (NNIPS), Krishna Galli, Lalitpur, Kathmandu, Nepal

[c]Department of Dentistry, Institute of Medicine, Tribhuhvan University. Kathmandu, Nepal

[d]Department of Advanced Oral Sciences and Therapeutics, University of Maryland School of Dentistry, Baltimore, MD, United States

Emails: Khatry SK (skhatry@wlink.com.np), Agrawal NK (cdrnitin@gmail.com), Katz J (jkatz1@jhu.edu), LeClerq SC (sleclerq@mos.com.np), Rai B (bholarai19@gmail.com), Reynolds MA (MReynolds@umaryland.edu), Mullany LC (lmullany@jhu.edu)

Correspondence: Daniel J. Erchick, PhD, MPH. Department of International Health, Johns Hopkins Bloomberg School of Public Health, 615 N. Wolfe Street, Baltimore, MD, 21205, United States. derchick@jhu.edu

Word Count: 3,875

For peer review only

For peer review only - http://bmjopen.bmj.com/site/about/guidelines.xhtml

ABSTRACT

Objectives: Observational studies have identified associations between periodontitis and adverse pregnancy outcomes, but randomized controlled trials evaluating the efficacy of periodontal therapy have yielded inconsistent results. Few studies have explored relationships between gingival inflammation and these outcomes or been conducted in rural, low-income communities, where confounding risk factors differ from other settings.

Methods: We conducted a community-based, prospective cohort study with the aim of estimating associations between the extent of gingival inflammation in pregnant women and incidence of preterm birth in rural Nepal. Our primary exposure was gingival inflammation, defined as bleeding on probing (BOP) ≥10%, stratified by BOP <30% and BOP ≥30%. A secondary exposure, mild periodontitis, was defined as ≥2 interproximal sites with probing depth (PD) ≥4 mm (different teeth) or one site with PD≥5 mm. Our primary outcome was preterm birth (<37 weeks gestation). We used Poisson regression to model this relationship, adjusting for potential confounders.

Results: Of 1,394 participants, 554 (39.7%) had gingival inflammation, 54 (3.9%) mild periodontitis, and 197 (14.1%) delivered preterm. In the adjusted regression model, increasing extent of gingival inflammation was associated with a non-significant increase in risk of preterm birth (BOP ≥30% vs. no BOP: aRR 1.37, 95% CI: 0.81, 2.32). A secondary analysis, stratifying participants by when in pregnancy their oral health status was assessed, showed an association between gingival inflammation and preterm birth among women examined in their first trimester

For peer review only - http://bmjopen.bmj.com/site/about/guidelines.xhtml

(BOP ≥30% vs. no BOP: aRR 2.57, 95% CI: 1.11, 5.95), but not later in pregnancy (BOP ≥30% vs. no BOP: aRR 1.05, 95% CI: 0.52, 2.11).

Conclusions: Gingival inflammation in women examined early in pregnancy and poor oral hygiene behaviors were risk factors for preterm birth. Future studies should evaluate community-based oral health interventions that specifically target gingival inflammation, delivered early in or before pregnancy, on preterm birth.

Strengths and Limitations of this Study

- This study collected data through a large, prospective, community-based cohort in a rural, low-resource setting.
- The study population had low prevalence of some established confounders of the relationship of interest, which are common in other populations.
- Some clinical periodontal measures were not collected due to visit time constraints, for example, recession on interproximal sites or gingival index score.
- Preterm birth classification was based upon maternal self-report of last menstrual period (LMP) instead of the gold standard, ultrasound examination.

For peer review only

INTRODUCTION

Annually, 2.5 million babies die prior to 28 days of life, and preterm birth is the leading cause of these deaths.[1] Preterm newborns that survive are at substantial risk of mortality from other causes, long-term disabilities such as neurological and developmental impairments, and non-communicable diseases.[2] In low- and middle-income countries (LMICs), where the majority of preterm births occur, therapeutic interventions are often unavailable and difficult to scale up, especially in communities where many mothers deliver at home or in primary facilities without skilled care (e.g. South Asia).[3]

Periodontal disease includes several inflammatory conditions, typically initiated by oral bacteria, beginning with reversible accumulation of plaque and inflammation of gingival tissue (gingivitis) and progressing to irreversible breakdown of the supportive tissues of the teeth and tooth loss (periodontitis).[4] Onset of new and worsening of existing gingival inflammation during pregnancy are normal and well documented, peaking in the second or third trimester.[5, 6] Major physiological and hormonal changes occur during pregnancy with wide ranging effects on the body, including increased permeability of gingival capillaries, altered immune system activity, and shifts in composition of the sub-gingival microbiome, including proliferation of aggressive bacteria associated with periodontitis, such as *Porphyromonas gingivalis.*[7, 8]

Periodontal disease in pregnant women has been associated with preterm birth and other adverse pregnancy outcomes.[9-11] Randomized controlled trials (RCTs) evaluating the impact of periodontal therapy during pregnancy on adverse pregnancy outcomes have produced inconsistent results.[9, 12-18] One meta-analysis found a significant effect of periodontal therapy

For peer review only - http://bmjopen.bmj.com/site/about/guidelines.xhtml

among women at high risk of preterm birth.[19] Although the mechanisms underlying this observed association are unclear, hypotheses include hematogenic translocation of periodontal pathogens or their byproducts to the fetal-placental unit, or action of inflammatory mediators in the periodontium on levels of systemic inflammation.[20]

Alternatively, the observed relationship between periodontal disease in pregnant women and adverse pregnancy outcomes could be the result of unmeasured and uncontrolled confounding factors. Previously described confounders of this relationship, which are commonly controlled for in studies, include age, smoking, multiple birth, previous adverse pregnancy outcomes, ethnicity, and socio-economic factors.[11] Some studies, however, have proposed the possibility that a genetic inflammatory phenotype could be responsible for increased risk of periodontal disease, or failed periodontal theory, and adverse pregnancy outcomes, particularly spontaneous preterm birth.[21]

Few studies have evaluated whether gingival inflammation is associated with risk of preterm birth or other adverse pregnancy outcomes. Further, studies of this association have been nearly universally facility-based, whether in high- or low-income settings. Understanding this relationship from a population-based perspective in low resource communities can offer certain benefits from an epidemiological perspective. Many populations in LMICs have lower prevalence of important confounding factors of this relationship, such as smoking, alcohol use, and chronic diseases (e.g. hypertension or diabetes). Community-based studies can avoid selection bias associated with hospital-based studies, particularly in populations where home delivery remains common, as is the case in much of South Asia. Given these potential benefits,

For peer review only

we conducted a community-based, prospective cohort study to estimate the association between gingival inflammation and preterm birth among women in a rural community in the Terai (plains) region of Nepal.

For peer review only

For peer review only - http://bmjopen.bmj.com/site/about/guidelines.xhtml

METHODS

We conducted a community-based, prospective cohort study of maternal gingival inflammation and adverse pregnancy outcomes across nine village development committees (VDCs) in Sarlahi District, Nepal. Eligible participants included married pregnant women between the ages of 15 and 40 who were <26 weeks gestation at the time of enrollment. Participants were identified and determined eligible between January and November 2016 using the infrastructure of a large community-based randomized trial of topical applications for newborn massage, the Nepal Oil Massage Study (NOMS) (NCT01177111), which was actively enrolling a population-based sample of pregnant women in this study area.

For peer review only

Study visits

Study visits were conducted in participant homes because of the wide dispersion of households across this rural community and the impracticality of bringing participants to a central location. Data on participant demographics, vital signs and morbidities during pregnancy, oral hygiene practices, care-seeking, and knowledge, and other characteristics were collected through a series of questionnaires administered over several visits during the course of pregnancy. Data collection teams were notified of the birth outcome by a locally resident study staff member, and the date of birth and other data concerning the mother and newborn were collected as soon as possible after delivery.

Oral health examinations were performed by five auxiliary nurse midwives who were trained for the purpose of this study. Their training lasted 3-4 weeks and included classroom instruction and practice of periodontal techniques conducted by an experienced dentist (NKA) at the Department

For peer review only - http://bmjopen.bmj.com/site/about/guidelines.xhtml

of Dentistry, Institute of Medicine, Tribhuvann University, Kathmandu, Nepal. We estimated the validity of PD measurements of the auxiliary nurse midwives relative to the dentist (NKA), finding percent agreement, weighted kappa scores, and intraclass correlation coefficients, with an allowance of PD ±1 mm, exceeded 99%, 0.7, and 0.9, respectively.[22]

Periodontal measurements

Auxiliary nurse midwives used portable dental equipment to conduct a full mouth examination. Periodontal measurements were made using a color Williams probe (Hu-Friedy, Chicago, IL, USA). PD was measured on six sites per tooth (disto-, mid-, and mesial- aspects of buccal and lingual surfaces) and CEJ-GM distance on two sites per tooth (mid- buccal and lingual aspects), excluding third molars. Presence or absence of BOP was recorded for any buccal or lingual probing site of each tooth. PD values were recorded in millimeters rounded to the next higher whole number. CEJ-GM distances were recorded similarly. If the free gingiva was coronal to the CEJ, the CEJ-GM measurement was recorded as 0. Clinical attachment loss (CAL) was calculated by summing PD and CEJ-GM distances.

Outcome/exposure definitions

Our primary exposure definition, gingival inflammation, was defined according to a classification scheme developed by the American Academy of Periodontology (AAP) and European Federation of Periodontology to update the 1999 classification of periodontal diseases and conditions.[23] Clinical health was defined as all sites PD ≤3 mm and BOP <10%.[24, 25] Gingival inflammation was defined as BOP ≥10%, stratified as localized gingival inflammation (BOP 10%-30%) and generalized gingival inflammation (BOP ≥30%).[24-29]

For peer review only - http://bmjopen.bmj.com/site/about/guidelines.xhtml

We defined secondary exposure definitions for mild and moderate periodontitis based upon a modified version of Centers for Disease Control and AAP updated 2012 case definitions for population-based surveillance of periodontitis.[28, 30] Mild periodontitis was defined as ≥2 interproximal sites with PD≥4 mm (not on the same tooth) or one site with PD≥5 mm. Moderate periodontitis was defined as ≥2 interproximal sites with PD≥5 mm (not on the same tooth). CAL was not included in these definitions as we did not collect this measure at interproximal sites to shorten the visit time; however, clinical attachment level and probing depth are widely considered equivalent measures of periodontitis in younger adults.[31]

Gestational age was calculated using the last menstrual period (LMP) method as recalled by the mother at 5-weekly pregnancy surveillance home visits. Our 5-weekly pregnancy surveillance approach has several benefits, including short recall (≤5 weeks) and pregnancy testing, as compared to traditional LMP approaches, which often rely on LMP recall later in pregnancy or at time of delivery. Preterm birth was defined as a live birth or stillbirth <37 completed weeks of gestation at the time of delivery.

Statistical analysis

Bivariate analyses between participant characteristics and the outcome, preterm birth, were evaluated using t-tests and Poisson regression for continuous and binary/categorical variables, respectively. We calculated unadjusted and adjusted relative risks (RR & aRR) of preterm birth and associated 95% confidence internals (CI) using Poisson regression with robust variance, which was used due to occurrence of convergence issues with other regression methods. Given

For peer review only - http://bmjopen.bmj.com/site/about/guidelines.xhtml

our sample size (n=1,394) and assuming a power of 80%, type I error of 0.05, and our pre-study estimates of population prevalence of preterm birth (17%) and gingival inflammation (40%), we estimated that we could detect a relative risk of preterm birth of roughly >1.5.

Multivariable models were constructed by sequentially adding groups of covariates, beginning with maternal characteristics, oral hygiene behaviors, and socioeconomic factors. Covariates associated with preterm birth at the p<0.10 level in bivariate analyses were considered in these regression models. Additional variables, known through previous studies to be confounders of the periodontal disease and preterm birth relationship, were also included in regression models (including age, ethnicity, body mass index (BMI), primiparity, multiple births, and socioeconomic variables (i.e., literacy, education, and indicators of household wealth).

In an effort to remove a possibly attenuating effect of including cases of transient pregnancy-induced gingival inflammation, we examined the relationship stratified by timing of oral exam. Women were categorized into two trimester groups (<13 weeks and ≥13 to <26 weeks gestation). Similar to the primary analysis, for each trimester group we conducted adjusted relative risks (RR & aRR) of preterm birth and associated 95% confidence internals (CI) using Poisson regression with robust variance.

All statistical analyses were performed in STATA 14.2 (StataCorp, College Station, TX, USA). All participants provided written consent for this study. Patients or the public were not involved in the design, or conduct, or reporting, or dissemination plans of our research. The study received ethical approval from the Institutional Review Board at Johns Hopkins Bloomberg School of

For peer review only - http://bmjopen.bmj.com/site/about/guidelines.xhtml

Public Health (Baltimore, USA) and the Ethical Review Board of the Nepal Health Research Council (Kathmandu, Nepal).

For peer review only

For peer review only - http://bmjopen.bmj.com/site/about/guidelines.xhtml

RESULTS

Participant characteristics

Between January 11, 2016, and November 26, 2016, among 2,821 pregnancies in the study area, 2,291 (81.2%) participants were identified by the parent trial as eligible for enrollment (<26 weeks gestation) (Figure 1). Among eligible participants, 1,478 (64.5%) were enrolled and six refused participation (0.3%). Another 807 (35.2%) were not visited for the purposes of the study because the eligible participants in the study area initially exceeded the capacity of the auxiliary nurse midwives to enroll women before their gestational age exceeded the exclusion criterion of <26 weeks. For logistical reasons, enrollment of women earlier in pregnancy was prioritized at the start of study enrollment, while enrollment of women later in pregnancy was prioritized towards the end of study enrollment. However, among eligible women, the included and excluded participant groups did not differ by age, education, or socio-economic factors.

Birth outcomes for the 1,474 women followed until the end of pregnancy were recorded as 1,345 single live births, 14 twin live births, 33 single stillbirths, 1 twin stillbirth, 1 set of twins where 1 was live born and 1 stillborn, 74 miscarriages, and 6 abortions. As our primary outcome was preterm birth, pregnancy was selected as the unit of analysis (and, therefore, twins were counted only once). There were 1,394 total pregnancies for analysis, of which 197 (14.1%) were preterm. Preterm deliveries included 163 (11.7%) moderate preterm (<37 to ≥32 weeks), 27 (1.9%) very preterm (<32 to ≥28 weeks), and 7 (0.5%) extremely preterm (<28 weeks). Over three-quarters of participants (n=1,053, 75.5%) were full term (≥37 weeks) and 144 (10.3%) were post-term (≥42 weeks).

For peer review only - http://bmjopen.bmj.com/site/about/guidelines.xhtml

Baseline demographic, periodontal, oral hygiene, and dental health care seeking characteristics of participants by extent of gingival inflammation were previously reported.[32] At enrollment, mean gestation was 14.5 (SD: 4.3) weeks (range: 6.4 to 27.7 weeks), with 574 women in their first trimester and 820 in their second. Participant age averaged 23.0 ± 4.6 years, and risk of preterm birth was higher (RR 1.93, CI: 1.01, 3.72) for women ≥35 years relative to those 18-35 years (Table 1). Several known confounders of the gingival inflammation and preterm birth relationship had a reported prevalence near 0% in this study population, including smoking and other tobacco use, alcohol use, and hypertension.

For peer review only

Table 1: Participant characteristics at enrollment (n=1,394)

Characteristic	All	Term	Preterm	Mean difference / RR (95% CI)*
**Age**				
Year (mean ± SD)	23.0 ± 4.6	23.0 ± 4.6	23.0 ± 5.1	0.02 (-0.68, 0.72)
**Age (years)**				
<18	151 (10.8)	124 (10.4)	27 (13.7)	1.33 (0.92, 1.93)
18-<35	1216 (87.2)	1053 (88.0)	163 (82.7)	Ref
≥35	27 (1.9)	20 (1.7)	7 (3.6)	**1.93 (1.01, 3.72)**
**Ethnic group**				
Hills (Pahadi)	101 (7.3)	89 (7.4)	12 (6.1)	Ref
Plains (Madeshi)	1292 (92.7)	1107 (92.6)	185 (93.9)	1.21 (0.70, 2.08)
**Height**				
Cm (mean ± SD)	150.8 ± 5.5	150.9 ± 5.6	150.1 ± 5.1	-0.80 (-1.63, 0.03)
**Weight**				
Kg (mean ± SD)	45.8 ± 7.0	46.0 ± 7.1	44.3 ± 5.8	**-1.67 (-2.72, -0.62)**
**BMI**				
Underweight (<18.5 kg)	418 (30.0)	353 (29.5)	65 (33.0)	1.09 (0.83, 1.44)
Normal weight (18.5-<25 kg)	893 (64.1)	766 (64.0)	127 (64.5)	Ref
Overweight or obese (≥25 kg)	83 (6.0)	78 (6.5)	5 (2.5)	0.42, (0.18, 1.01)
**High blood pressure**				
No	1384 (99.3)	1188 (99.2)	196 (99.5)	Ref
Yes	10 (0.7)	9 (0.8)	1 (0.5)	0.71 (0.11, 4.56)
**Gravidity**				
First pregnancy	395 (28.3)	332 (27.7)	63 (32.0)	1.23 (0.92, 1.63)
1-3 previous pregnancies	838 (60.1)	729 (60.9)	109 (55.3)	Ref
≥4 pregnancies	161 (11.5)	136 (11.4)	25 (12.7)	1.19 (0.80, 1.78)
**Urinary or vaginal infection+**				
No	1097 (78.7)	942 (78.7)	155 (78.7)	Ref
Yes	297 (21.3)	255 (21.3)	42 (21.3)	1.00 (0.73, 1.37)

For peer review only - http://bmjopen.bmj.com/site/about/guidelines.xhtml

**Literacy**				
No	751 (53.9)	638 (53.3)	113 (57.4)	Ref
Yes	643 (46.1)	559 (46.7)	84 (42.6)	0.87 (0.67, 1.13)
**Education (years)**				
0	751 (53.9)	634 (53.0)	117 (59.4)	Ref
1-9	386 (27.7)	343 (28.7)	43 (21.8)	**0.72 (0.52, 0.99)**
≥10	256 (18.4)	219 (18.3)	37 (18.8)	0.93 (0.66, 1.31)
**Electricity**				
No	114 (8.2)	97 (8.1)	17 (8.6)	Ref
Yes	1279 (91.8)	1099 (91.9)	180 (91.4)	0.94 (0.60, 1.49)
**House construction material**				
None, thatch, sticks, or bamboo	858 (61.6)	749 (62.6)	109 (55.3)	Ref
Wood planks, bricks, or stone	535 (38.4)	447 (37.4)	88 (44.7)	**1.29 (1.00, 1.68)**
**House roof material**				
None, plastic, thatch, or grass	110 (7.9)	92 (7.7)	18 (9.1)	Ref
Tile/tin/concrete	1283 (92.1)	1104 (92.3)	179 (90.9)	0.85 (0.55, 1.33)
**Latrine**				
No latrine	601 (43.1)	515 (43.1)	86 (43.7)	Ref
Brick, concrete, or pit latrine	792 (56.9)	681 (56.9)	111 (56.3)	0.98 (0.75, 1.27)

Data presented as No. (%) unless otherwise noted

* T-test or relative risk and 95% confidence interval as appropriate

+ Self-reported symptoms of painful urination or foul smelling vaginal discharge during pregnancy

For peer review only

For peer review only - http://bmjopen.bmj.com/site/about/guidelines.xhtml

According to our definition, 554 (39.7%) participants had gingival inflammation and 840 (60.3%) were healthy (Table 2). Of those with gingival inflammation, 445 (80.3%) had localized gingival inflammation (BOP <30%) and 109 (19.7%) generalized gingival inflammation (BOP ≥30%). Most participants (n=1,105, 79.3%) had at least one site with BOP. According to our secondary exposure definitions, 54 participants (3.9%) had mild and 4 (0.3%) moderate periodontitis. More participants (n=120, 8.6%) had ≥1 site with PD ≥4 mm. Mean CAL was equivalent to mean PD, with nearly all of CAL ≥4 mm due to pocketing secondary to gingival enlargement, indicating very little recession of the gingiva in this population.

For peer review only

For peer review only - http://bmjopen.bmj.com/site/about/guidelines.xhtml

Table 2: Periodontal disease status (n=1,394)

Characteristic	All	Term	Preterm	Rate of preterm
**Health and gingival inflammation**				
Health (All sites PD ≤3 mm and BOP <10%)	840 (60.3)	727 (60.7)	113 (57.4)	13.5%
Gingival inflammation (BOP ≥10% and/or PD ≥4 mm)	554 (39.7)	470 (39.3)	84 (42.6)	15.2%
- Localized gingival inflammation (BOP <30%)	445 (80.3)	379 (80.6)	66 (78.6)	14.8%
- Proportion with no sites PD ≥4 mm	354 (79.6)	299 (78.9)	55 (83.3)	15.5%
- Proportion with ≥1 sites PD ≥4 mm	91 (20.5)	80 (21.2)	11 (16.7)	12.1%
- Generalized gingival inflammation (BOP ≥30%)	109 (19.7)	91 (19.4)	18 (21.4)	16.5%
- Proportion with no sites PD ≥4 mm	80 (73.4)	66 (72.5)	14 (77.8)	17.5%
- Proportion with ≥1 sites PD ≥4 mm	29 (26.6)	25 (27.5)	4 (22.2)	13.8%
**Bleeding on probing (BOP)**				
Percent of sites BOP (mean ± SD)	10.2 ± 12.3	10.1 ± 12.4	10.6 ± 12.0	14.1%*
No sites BOP	289 (20.7)	249 (20.8)	40 (20.3)	13.8%
≥1 site BOP	1,105 (79.3)	949 (79.2)	157 (79.7)	14.2%
≥1 site BOP & BOP <10%	601 (43.1)	521 (43.5)	80 (40.6)	13.3%
BOP ≥10% & BOP <30%	395 (28.3)	336 (28.1)	59 (30.0)	14.9%
BOP ≥30%	109 (7.8)	91 (7.6)	18 (9.1)	16.5%
**Probing depth (PD)**				
Mean PD (mm) (mean ± SD)	1.7 ± 0.3	1.7 ± 0.3	1.7 ± 0.3	14.1%*
Mean PD at direct sites (mm) (mean ± SD)	1.5 ± 0.3	1.5 ± 0.3	1.5 ± 0.2	14.1%*
Percent of sites PD ≥4 mm (mean ± SD)	0.2 ± 1.0	0.2 ± 1.0	0.1 ± 0.9	14.1%*
≥1 site PD ≥4 mm	120 (8.6)	105 (8.8)	15 (7.6)	12.5%
**Clinical attachment loss (CAL)**				
Mean CAL at direct sites (mm) (mean ± SD)	1.7 ± 0.3	1.7 ± 0.3	1.7 ± 0.3	14.1%*
≥1 site recession ≥1 mm	173 (12.4)	154 (12.9)	19 (9.6)	11.0%
≥1 site CAL ≥4 mm	196 (14.1)	172 (14.4)	24 (12.2)	12.2%
Percent of CAL ≥4 mm due to pocketing (mean ± SD)	99.6 ± 1.5	99.6 ± 1.5	99.8 ± 0.9	14.1%*
Percent of CAL ≥4 mm due to recession (mean ± SD)	0.4 ± 1.5	0.4 ± 1.5	0.2 ± 0.9	14.1%*

Data presented as No. (%) unless otherwise noted

* Preterm birth rate among all participants

For peer review only - http://bmjopen.bmj.com/site/about/guidelines.xhtml

Several poor oral hygiene behaviors were associated with increased risk of preterm birth, including extended teeth cleaning time (≥30 mins vs. <5 mins: RR 2.48, 95% CI: 1.41, 4.39), use of a non-traditional dentifrice (i.e., sand, ash, oil, or gul) (RR 2.22, 95% CI: 1.44, 3.42). Teeth cleaning episodes per week (≥14 vs. <7 times: RR 0.55, 95% CI: 0.29, 1.02) was associated with decreased risk of preterm birth. Risk of preterm birth increased with a dose response pattern for women who practiced multiple poor oral hygiene behaviors, defined as extended cleaning time per episode (≥5 minutes), infrequent cleaning episodes (<7 times per week), and use of a traditional dentifrice. Incidence of preterm birth was 17/208 (8.2%) for women with no poor behaviors, 155/1,098 (14.1%) for women with one, and 25/88 (28.4%) for those with two.

Primary analysis: Association between maternal gingival inflammation and preterm birth

Incidence of preterm birth by periodontal status was 113/840 (13.5%) for health and 84/554 (15.2%) for gingival inflammation. Incidence of preterm birth increased from 120/890 (13.5%) for women with either health (no sites BOP) or BOP <10% to 59/395 (14.9%) for women with localized gingival inflammation (BOP ≥10% & <30%) to 18/109 (16.5%) for women with generalized gingival inflammation (BOP ≥30%).

Our binary definition of gingival inflammation (BOP ≥10%) was not associated with preterm birth in the crude model (RR 1.13, 95% CI: 0.87, 1.46) or after adjusting for confounders (aRR 1.16, 95% CI: 0.90, 1.51). In the final adjusted model, increasing extent of gingival inflammation was associated with a non-significant increase in risk of preterm birth (BOP ≥30% vs. no BOP: aRR 1.37, 95% CI: 0.81, 2.32) (Table 3). In this model, several oral hygiene behaviors were associated with preterm birth. Extended cleaning time (≥30 mins vs. <5 mins: aRR 2.44, 95% CI:

For peer review only - http://bmjopen.bmj.com/site/about/guidelines.xhtml

1.34, 4.43), use of a traditional dentifrice (n=53, 3.9%) (aRR 2.17, 95% CI: 1.39, 3.38), and fewer teeth cleaning episodes per week (≥14 times vs. 1-6 times: aRR 0.46, 95% CI: 0.25, 0.84) were associated with increased risk of preterm birth.

For peer review only

For peer review only - http://bmjopen.bmj.com/site/about/guidelines.xhtml

Table 3: Adjusted relative risks (aRR) between maternal gingival inflammation and preterm birth

Characteristic	Unadjusted model (n=1,394)	Preterm birth aRR (95% CI) Model 1 (n=1,393)	Model 2 (n=1,358)	Final model (n=1,357)
**Gingival inflammation**				
No sites BOP	Ref	Ref	Ref	Ref
≥1 site BOP & BOP <10%	0.96 (0.68, 1.37)	0.99 (0.70, 1.40)	1.08 (0.76, 1.54)	1.07 (0.75, 1.51)
BOP ≥10% & BOP <30%	1.08 (0.74, 1.57)	1.11 (0.77, 1.61)	1.19 (0.82, 1.73)	1.17 (0.80, 1.69)
BOP ≥30%	1.19 (0.72, 1.99)	1.24 (0.74, 2.08)	1.34 (0.79, 2.28)	1.37 (0.81, 2.32)
**Age (years)**				
<18		1.21 (0.80, 1.85)	1.17 (0.77, 1.76)	1.21 (0.80, 1.84)
18-<35		Ref	Ref	Ref
≥35		**2.17 (1.14, 4.11)**	1.82 (0.95, 3.49)	1.65 (0.88, 3.10)
**Pahadi/Madeshi**				
Pahadi		Ref	Ref	Ref
Madeshi		1.13 (0.65, 1.97)	0.95 (0.55, 1.66)	0.97 (0.55, 1.70)
**BMI (kg)**				
Underweight (<18.5)		1.10 (0.83, 1.45)	1.11 (0.85, 1.47)	1.15 (0.87, 1.52)
Normal weight (18.5-<25)		Ref	Ref	Ref
Overweight or obese (≥25)		0.45 (0.18, 1.08)	0.49 (0.20, 1.18)	0.47 (0.20, 1.13)
**Primiparous**				
No		Ref	Ref	Ref
Yes		1.17 (0.86, 1.60)	1.25 (0.92, 1.71)	1.26 (0.91, 1.75)
**Multiple births**				
No		Ref	Ref	Ref
Yes		**4.46 (2.88, 6.92)**	**3.76 (2.46, 5.75)**	**3.38 (2.09, 5.46)**
**Teeth cleaning time per episode (mins)**				
<5			Ref	Ref
5-<30			**1.77 (1.07, 2.94)**	**1.83 (1.11, 3.04)**
≥30			**2.37 (1.31, 4.27)**	**2.44 (1.34, 4.43)**
**Teeth cleaning episodes per week**				
1-6			Ref	Ref

For peer review only - http://bmjopen.bmj.com/site/about/guidelines.xhtml

7-13	0.64 (0.39, 1.05)	**0.62 (0.39, 1.00)**
≥14	**0.48 (0.26, 0.89)**	**0.46 (0.25, 0.84)**
**Use of traditional dentifrice**		
No	Ref	Ref
Yes	**2.13 (1.37, 3.31)**	**2.17 (1.39, 3.38)**
**Literacy**		
No		Ref
Yes		1.51 (0.85, 2.66)
**Education (years)**		
0		Ref
1-9		**0.50 (0.27, 0.92)**
≥10		0.65 (0.35, 1.21)
**House construction material**		
None, plastic, thatch, or grass		Ref
Wood planks, bricks, or stone		**1.47 (1.11, 1.93)**
**Roof construction material**		
None, plastic, thatch, or grass		Ref
Tin, tile, or concrete		0.85 (0.55, 1.33)

For peer review only

Secondary analysis: Stratification by gestational age at periodontal examination

In a secondary analysis, participants were stratified by their trimester at the time of the periodontal examination (1st trimester: n=574; 2nd trimester: n=820). Among women with dental exams in the first trimester, incidence of preterm birth increased from 47/381 (12.3%) for women with either health (no sites BOP) or BOP <10% to 28/158 (17.7%) for women with localized gingival inflammation (BOP ≥10% & <30%) to 8/35 (22.9%) for women with generalized gingival inflammation (BOP ≥30%). Our binary definition of gingival inflammation (BOP ≥10%) in the first trimester was associated with preterm (aRR 1.62, 95% CI: 1.08, 2.42), but not in the second trimester (aRR 0.92, 95% CI: 0.65, 1.30), after adjusting for confounders. In the final adjusted model stratified by trimester, there was a positive relationship between gingival inflammation and risk of preterm birth among women in the first trimester (BOP ≥30% vs. no BOP: aRR 2.57, 95% CI: 1.11, 5.95), but not among women in their second trimester (BOP ≥30% vs. no BOP: aRR 1.05, 95% CI: 0.52, 2.11) (Table 4).

Our secondary exposure definition for mild periodontitis was not associated with preterm in a binary comparison or the crude or adjusted regression models for our primary or secondary analyses. These analyses were not conducted for moderate periodontitis given the low prevalence of the condition in this study population.

For peer review only - http://bmjopen.bmj.com/site/about/guidelines.xhtml

Table 4: Adjusted relative risks (aRR) between maternal gingival inflammation and preterm birth stratified by trimester at periodontal examination visit

	Preterm birth aRR (95% CI)	
**Characteristic**	**Women in 1st trimester (n=559)**	**Women in 2nd trimester (n=798)**
**Gingival inflammation**		
No sites BOP	Ref	Ref
≥1 site BOP & BOP <10%	1.51 (0.81, 2.84)	0.93 (0.60, 1.44)
BOP ≥10% & BOP <30%	**1.91 (1.00, 3.63)**	0.88 (0.55, 1.42)
BOP ≥30%	**2.57 (1.11, 5.95)**	1.05 (0.52, 2.11)
**Age (years)**		
<18	1.06 (0.52, 2.18)	1.42 (0.85, 2.36)
18-<35	Ref	Ref
≥35	0.97 (0.28, 3.38)	**2.42 (1.14, 5.14)**
**Pahadi/Madeshi**		
Pahadi	Ref	Ref
Madeshi	1.15 (0.46, 2.90)	0.82 (0.42, 1.60)
**BMI (kg)**		
Underweight (<18.5)	1.44 (0.95, 2.18)	0.96 (0.65, 1.42)
Normal weight (18.5-<25)	Ref	Ref
Overweight or obese (≥25)	0.72 (0.19, 2.78)	0.33 (0.11, 1.06)
**Primiparous**		
No	Ref	Ref
Yes	1.41 (0.84, 2.34)	1.13 (0.74, 1.72)
**Multiple births**		
No	Ref	Ref
Yes	2.57 (0.92, 7.15)	**4.38 (2.57, 7.47)**
**Teeth cleaning time per episode (mins)**		
<5	Ref	Ref
5-<30	1.40 (0.71, 2.75)	**2.42 (1.13, 5.19)**
≥30	2.06 (0.92, 4.57)	**2.93 (1.19, 7.23)**
**Teeth cleaning episodes per week**		
1-6	Ref	Ref
7-13	0.87 (0.39, 1.93)	**0.49 (0.28, 0.85)**
≥14	0.49 (0.18, 1.31)	**0.46 (0.22, 0.95)**
**Use of traditional dentifrice**		
No	Ref	Ref
Yes	1.61 (0.63, 4.10)	**2.58 (1.56, 4.26)**
**Literacy**		
No	Ref	Ref
Yes	1.97 (0.78, 4.96)	1.25 (0.60, 2.61)
**Education (years)**		
0	Ref	Ref
1-9	**0.34 (0.13, 0.89)**	0.67 (0.31, 1.45)

For peer review only

For peer review only - http://bmjopen.bmj.com/site/about/guidelines.xhtml

≥10	0.41 (0.15, 1.11)	0.93 (0.41, 2.10)
**House construction material**		
None, plastic, thatch, or grass	Ref	Ref
Wood planks, bricks, or stone	1.33 (0.84, 2.11)	**1.57 (1.09, 2.25)**
**Roof construction material**		
None, plastic, thatch, or grass	Ref	Ref
Tin, tile, or concrete	0.80 (0.39, 1.65)	0.90 (0.51, 1.59)

For peer review only

DISCUSSION

In our community-based, prospective cohort study, gingival inflammation was an independent risk factor for preterm birth among women examined during their first, but not second, trimester, after adjusting for potential confounding risk factors. Our finding suggests that gingival infection and inflammatory burden early in pregnancy, potentially originating prior to conception, could be responsible for the observed relationship, which becomes obscured in the second trimester by the presence of women with initially healthy periodontal conditions who develop pregnancy-induced gingival inflammation. This result is consistent with studies that have reported that active, and especially progressive, periodontal infection is most harmful early in pregnancy.[13] This also supports the hypothesis, proposed to explain the mixed results of RCTs of this association, that intervening on women with periodontal disease later in pregnancy, or even after conception, may be too late to affect the proposed causal pathway.[33]

Most previous studies have considered the relationship between varying definitions and severities of periodontitis and preterm birth. A meta-analysis of seven cohort studies estimated a pooled relative risk of preterm of 1.70 (1.03, 2.81) for pregnant women with periodontitis versus health.[10] Prevalence of periodontitis in our study population was low, although not dissimilar to previous surveys of periodontal conditions among this age group in Nepal.[34] Additional data on the clinical periodontal characteristics and oral hygiene behaviors of women in our study are published in detail elsewhere.[32] The low prevalence of periodontitis in this population is likely attributable to the population's low age (median 22.2, IQR: 19.7, 25.5; mean 23.0, SD: 4.6) and absence of other common risk factors for the condition. An unpublished review of community-based studies of the periodontal disease and preterm birth relationship in low- and middle-income countries conducted by our study team found mean maternal ages ranging from 26 to 29.

For peer review only - http://bmjopen.bmj.com/site/about/guidelines.xhtml

Unlike the majority of studies of this association, particularly in high-income countries, the prevalence of important confounders, such as smoking, alcohol use, and morbidities associated with chronic disease, were nearly absent in our study population. An association between periodontitis and preterm birth may have been seen in our population had there been a higher prevalence and severity of periodontitis, as would be expected in an older population.

Few observational or interventional studies have considered gingival inflammation as the primary exposure definition when investigating the relationship between periodontal disease and preterm birth. A cohort study by Kruse et al. (2008) found an association between gingivitis and high risk of preterm birth among women without periodontitis in a hospital setting in Germany.[35] A trial by Lopez et al. (2005) of women with gingivitis in a hospital setting in Santiago, Chile, demonstrated a significantly higher risk of preterm low birth weight among women with gingivitis who received periodontal treatment after delivery compared to those that received treatment during pregnancy (<28 weeks) (aOR: 2.76, 95% CI: 1.29, 5.88).[36] Gingival inflammation requires less intensive treatment than periodontitis, and can include improved oral hygiene, use of an antiseptic oral rinse, and periodontal therapy, interventions that could be feasibly delivered in a rural community setting. While antiseptic oral rinses are known for their ability to reduce plaque and control inflammation, their action on pregnancy outcomes is underexplored.[37, 38] However, Jeffcoat et al. (2011) found a reduction in preterm birth in a high-risk population with the use of cetylpyridinium chloride oral rinse intervention.[39]

Extent and severity of gingival inflammation, independent of traditional clinical measures of periodontitis, such as CAL or PD, have been associated with magnitude of bacteremia.[40]

For peer review only - http://bmjopen.bmj.com/site/about/guidelines.xhtml

Transient bacteremia, facilitated by ulceration, inflammation, and the increased vascular permeability of the gingiva, could pose increased risk for adverse pregnancy outcomes.[41] In this case, treatment of traditional clinical measures of periodontitis – without reducing infection, inflammation, and, potentially, the presence of specific harmful pathogens – could fail to disrupt the proposed causal pathway between exposure and outcome. Further, if such bacteremia were to occur early in pregnancy, treatment of clinical conditions later in pregnancy would be too late to eliminate exposure of the placenta and/or fetus to these pathogens, and hence would not decrease of risk of adverse pregnancy outcomes.[41]

Poor oral hygiene behaviors, including extended teeth cleaning time (≥5 mins), infrequent teeth cleaning (<7 times per week), and use of traditional dentifrice (i.e., sand, ash, oil, or gul) or datiwan, were also associated with increased risk of preterm birth in our analysis. Studies have shown that the mechanical manipulation involved in brushing, particularly frequent or forceful brushing or use of brushes with hard filaments, can lead to gingival abrasion, recession, and bacteremia.[42-44] Some have posited that bacteremia caused by mechanical manipulation of the gingiva involved in periodontal therapy may increase risk of adverse pregnancy outcomes, attenuating observation of any true effect in interventional studies of this association.[33] More research is needed to understand the extent to which poor oral hygiene behaviors in pregnant women, including common traditional practices, such as datiwan use, could – via local inflammatory responses or increased access of periodontal bacteria or their byproducts to the bloodstream – initiate a cascade of effects that ultimately influences the pregnancy outcome.

For peer review only - http://bmjopen.bmj.com/site/about/guidelines.xhtml

An important strength of our study was that we utilized a population-based sample, compared to previous observational studies of this relationship, which were primarily conducted in health facility settings, introducing risk of selection bias associated with the likelihood of women in the target population delivering in a particular facility. The potential for selection bias to influence the measure of association in a facility-based study is especially high in populations where a substantial proportion of women deliver at home, as is the case in this area of rural Nepal, where roughly half of women deliver in a facility. In an unpublished review conducted by our study team, we found one study of this association, by Mobeen et al. (2008), that used a population-based sample.[45] Conducted in a periurban area of Hyderabad, Pakistan, the authors reported significant associations of varying degrees between measures of periodontal disease and neonatal death, perinatal death, and stillbirth.[45]

A limitation of this study was the collection of clinical recession measures from only the direct buccal and lingual surfaces. In the absence of these data, we likely underestimated the burden of periodontal disease among pregnant women in this study, potentially attenuating our measure of association. We were unable to control for some confounders of this relationship, including previous preterm birth, certain chronic diseases (e.g., diabetes), and we only used a proxy (self-reported symptoms) for urinary tract and vaginal infections. Conducting periodontal exams in participant homes, where light and other conditions are variable, may have introduced a level of measurement error in our exposure that could not be quantified or controlled for given the setting and logistical constraints of our study. Lastly, preterm birth was based upon maternal self-report of last menstrual period (LMP) instead of ultrasound examination. If our LMP estimates were less accurate among women examined later in pregnancy, due to longer maternal recall relative

For peer review only - http://bmjopen.bmj.com/site/about/guidelines.xhtml

to women examined earlier in pregnancy, this could have resulted in non-differential misclassification of our binary outcome, preterm birth, which would tend to attenuate observation of a true relationship among women examined in the second trimester.

CONCLUSION

Our study identified gingival inflammation as an independent risk factor for preterm birth among women examined early in pregnancy. Women non-compliant with oral hygiene care recommendations were at substantially increased risk of preterm birth. These findings highlight the importance of adherence to proper home oral self-care in pregnant women and women expecting to become pregnant. Oral health policies and education programs should include tailored approaches to encourage improved self-care practices in this important population. Future studies should evaluate the effectiveness of community-based oral health interventions that specifically target gingival inflammation – delivered to women early in or prior to pregnancy – on the incidence of preterm birth and other adverse pregnancy outcomes in low-income countries with high risk for these outcomes.

For peer review only

For peer review only - http://bmjopen.bmj.com/site/about/guidelines.xhtml

Acknowledgements:
The authors thank the auxiliary nurse midwives who conducted the periodontal examinations: Kaushila Chaudhary, Lalita Lama, Gayatri Mahat, Shanti Sharma, and Santoshi Kumari. Thank you to Roshan Chaudhary for serving as the laboratory technician. We also wish a special thank you to the women from Sarlahi District, Nepal, for participating in this study.

This study was carried out in close collaboration with our implementing partner, Nepal Netra Jyoti Sangh, under the auspices of the Social Welfare Council of the Government of Nepal.

Competing interests: The authors declare that they have no competing interests.

Funding source: This work was supported by the National Institute for Child Health and Development [HD060712] and the Bill & Melinda Gates Foundation [OPP1084399, OPP1131701].

Trial registration: ClinicalTrials.gov Identifier: NCT01177111 (Nepal Oil Massage Study) and NCT02788786 (Pilot Trial).

Patients or public involvement: Patients or the public were not involved in the design, or conduct, or reporting, or dissemination plans of our research.

Data availability: All data files, codebooks, and related manuscripts are available from the JHU Data Archive (https://doi.org/10.7281/T1/ZPGBJW).

Abbreviations:

Adjusted relative risk (aRR)

Bleeding on probing (BOP)

Body mass index (BMI)

Cemento-enamel junction (CEJ)

Clinical attachment loss (CAL)

Confidence intervals (CI)

Gingival margin (GM)

For peer review only

For peer review only - http://bmjopen.bmj.com/site/about/guidelines.xhtml

Nepal Oil Massage Study (NOMS)

Relative risk (RR)

Probing depth (PD)

Standard deviation (SD)

Authors' contribution:

Dr. Erchick conceptualized and designed the study, developed field implementation protocols, led data collection in the field, conducted the analysis, and wrote the manuscript.

Dr. Khatry conceptualized and designed the study, oversaw field implementation and ensured quality data collection, and provided comments on the manuscript.

Dr. Agrawal conceptualized and designed the study, trained and oversaw the data collectors, and provided comments on the manuscript.

Dr. Katz conceptualized and designed the study, ensured quality data collection, advised on analytic approach, and provided comments on the manuscript.

Mr. LeClerq conceptualized and designed the study, supported overall implementation in the field, and provided comments on the manuscript.

Mr. Rai contributed to field implementation, ensured quality control of field procedures, and provided comments on the manuscript.

Dr. Reynolds conceptualized and designed the study, contributed to the analysis and interpretation of the results, and provided comments on the manuscript.

For peer review only - http://bmjopen.bmj.com/site/about/guidelines.xhtml

Dr. Mullany conceptualized and designed the study, obtained funding for the study, oversaw implementation of data collection, obtained ethical approvals, advised on analytic approach, and provided edits and comments on the manuscript.

All authors approved the final manuscript as submitted and agree to be accountable for all aspects of the work.

For peer review only

For peer review only - http://bmjopen.bmj.com/site/about/guidelines.xhtml

REFERENCES

1. United Nations Inter-agency Group for Child Mortality Estimation (UN IGME). Levels & Trends in Child Mortality. In: UNICEF, ed. New York, 2018.
2. Blencowe H, Cousens S, Chou D, et al. Born too soon: the global epidemiology of 15 million preterm births. *Reproductive health* 2013;10 Suppl 1:S2. doi: 10.1186/1742-4755-10-s1-s2 [published Online First: 2014/03/15]
3. Barros FC, Bhutta ZA, Batra M, et al. Global report on preterm birth and stillbirth (3 of 7): evidence for effectiveness of interventions. *BMC pregnancy and childbirth* 2010;10 Suppl 1:S3. doi: 10.1186/1471-2393-10-s1-s3 [published Online First: 2010/03/27]
4. Brown LJ, Loe H. Prevalence, extent, severity and progression of periodontal disease. *Periodontology 2000* 1993;2:57-71. [published Online First: 1993/06/01]
5. Loe H, Silness J. Periodontal disease in pregnancy. I. Prevalence and severity. *Acta odontologica Scandinavica* 1963;21:533-51. [published Online First: 1963/12/01]
6. Laine MA. Effect of pregnancy on periodontal and dental health. *Acta odontologica Scandinavica* 2002;60(5):257-64. [published Online First: 2002/11/07]
7. Figuero E, Carrillo-de-Albornoz A, Martin C, et al. Effect of pregnancy on gingival inflammation in systemically healthy women: a systematic review. *Journal of clinical periodontology* 2013;40(5):457-73. doi: 10.1111/jcpe.12053 [published Online First: 2013/04/06]
8. Wu M, Chen SW, Jiang SY. Relationship between gingival inflammation and pregnancy. *Mediators of inflammation* 2015;2015:623427. doi: 10.1155/2015/623427 [published Online First: 2015/04/16]
9. Offenbacher S, Katz V, Fertik G, et al. Periodontal infection as a possible risk factor for preterm low birth weight. *Journal of periodontology* 1996;67(10 Suppl):1103-13. doi: 10.1902/jop.1996.67.10s.1103 [published Online First: 1996/10/01]
10. Chambrone L, Guglielmetti MR, Pannuti CM, et al. Evidence grade associating periodontitis to preterm birth and/or low birth weight: I. A systematic review of prospective cohort studies. *Journal of clinical periodontology* 2011;38(9):795-808. doi: 10.1111/j.1600-051X.2011.01755.x [published Online First: 2011/06/29]
11. Ide M, Papapanou PN. Epidemiology of association between maternal periodontal disease and adverse pregnancy outcomes--systematic review. *Journal of periodontology* 2013;84(4 Suppl):S181-94. doi: 10.1902/jop.2013.134009 [published Online First: 2013/05/03]
12. Offenbacher S, Lieff S, Boggess KA, et al. Maternal periodontitis and prematurity. Part I: Obstetric outcome of prematurity and growth restriction. *Annals of periodontology / the American Academy of Periodontology* 2001;6(1):164-74. doi: 10.1902/annals.2001.6.1.164 [published Online First: 2002/03/13]
13. Offenbacher S, Boggess KA, Murtha AP, et al. Progressive periodontal disease and risk of very preterm delivery. *Obstetrics and gynecology* 2006;107(1):29-36. doi: 10.1097/01.aog.0000190212.87012.96 [published Online First: 2006/01/06]
14. Jeffcoat MK, Geurs NC, Reddy MS, et al. Periodontal infection and preterm birth: results of a prospective study. *Journal of the American Dental Association (1939)* 2001;132(7):875-80. [published Online First: 2001/08/02]
15. Michalowicz BS, Hodges JS, DiAngelis AJ, et al. Treatment of periodontal disease and the risk of preterm birth. *The New England journal of medicine* 2006;355(18):1885-94. doi: 10.1056/NEJMoa062249 [published Online First: 2006/11/03]
16. Offenbacher S, Beck JD, Jared HL, et al. Effects of periodontal therapy on rate of preterm delivery: a randomized controlled trial. *Obstetrics and gynecology* 2009;114(3):551-9. doi: 10.1097/AOG.0b013e3181b1341f [published Online First: 2009/08/25]

For peer review only - http://bmjopen.bmj.com/site/about/guidelines.xhtml

17. Offenbacher S, Lin D, Strauss R, et al. Effects of periodontal therapy during pregnancy on periodontal status, biologic parameters, and pregnancy outcomes: a pilot study. *Journal of periodontology* 2006;77(12):2011-24. doi: 10.1902/jop.2006.060047 [published Online First: 2007/01/11]
18. Iheozor-Ejiofor Z, Middleton P, Esposito M, et al. Treating periodontal disease for preventing adverse birth outcomes in pregnant women. *The Cochrane database of systematic reviews* 2017;6:Cd005297. doi: 10.1002/14651858.CD005297.pub3 [published Online First: 2017/06/13]
19. Kim AJ, Lo AJ, Pullin DA, et al. Scaling and root planing treatment for periodontitis to reduce preterm birth and low birth weight: a systematic review and meta-analysis of randomized controlled trials. *Journal of periodontology* 2012;83(12):1508-19. doi: 10.1902/jop.2012.110636 [published Online First: 2012/03/02]
20. Cetin I, Pileri P, Villa A, et al. Pathogenic mechanisms linking periodontal diseases with adverse pregnancy outcomes. *Reproductive sciences (Thousand Oaks, Calif)* 2012;19(6):633-41. doi: 10.1177/1933719111432871 [published Online First: 2012/03/17]
21. Jeffcoat MK, Jeffcoat RL, Tanna N, et al. Association of a common genetic factor, PTGER3, with outcome of periodontal therapy and preterm birth. *Journal of periodontology* 2014;85(3):446-54. doi: 10.1902/jop.2013.130006 [published Online First: 2013/06/29]
22. Erchick DJ, Agrawal NK, Khatry SK, et al. Feasibility of training community health workers to conduct periodontal examinations: A validation study in rural Nepal. 2020
23. Armitage GC. Development of a classification system for periodontal diseases and conditions. *Annals of periodontology / the American Academy of Periodontology* 1999;4(1):1-6. doi: 10.1902/annals.1999.4.1.1 [published Online First: 2000/06/23]
24. Chapple ILC, Mealey BL, Van Dyke TE, et al. Periodontal health and gingival diseases and conditions on an intact and a reduced periodontium: Consensus report of workgroup 1 of the 2017 World Workshop on the Classification of Periodontal and Peri-Implant Diseases and Conditions. *Journal of periodontology* 2018;89 Suppl 1:S74-s84. doi: 10.1002/jper.17-0719 [published Online First: 2018/06/22]
25. Trombelli L, Farina R, Silva CO, et al. Plaque-induced gingivitis: Case definition and diagnostic considerations. *Journal of periodontology* 2018;89 Suppl 1:S46-s73. doi: 10.1002/jper.17-0576 [published Online First: 2018/06/22]
26. Holtfreter B, Albandar JM, Dietrich T, et al. Standards for reporting chronic periodontitis prevalence and severity in epidemiologic studies: Proposed standards from the Joint EU/USA Periodontal Epidemiology Working Group. *Journal of clinical periodontology* 2015;42(5):407-12. doi: 10.1111/jcpe.12392 [published Online First: 2015/03/27]
27. Papapanou PN, Sanz M, Buduneli N, et al. Periodontitis: Consensus report of workgroup 2 of the 2017 World Workshop on the Classification of Periodontal and Peri-Implant Diseases and Conditions. *Journal of periodontology* 2018;89 Suppl 1:S173-s82. doi: 10.1002/jper.17-0721 [published Online First: 2018/06/22]
28. Tonetti MS, Greenwell H, Kornman KS. Staging and grading of periodontitis: Framework and proposal of a new classification and case definition. *Journal of periodontology* 2018;89 Suppl 1:S159-s72. doi: 10.1002/jper.18-0006 [published Online First: 2018/06/22]
29. World Health Organization. Epidemiology, etiology, and prevention of periodontal diseases. *World Health Organ Tech Rep Ser* 1978 [published Online First: 1978/01/01]
30. Eke PI, Page RC, Wei L, et al. Update of the case definitions for population-based surveillance of periodontitis. *Journal of periodontology* 2012;83(12):1449-54. doi: 10.1902/jop.2012.110664 [published Online First: 2012/03/17]
31. Page RC, Eke PI. Case definitions for use in population-based surveillance of periodontitis. *Journal of periodontology* 2007;78(7 Suppl):1387-99. doi: 10.1902/jop.2007.060264 [published Online First: 2007/08/19]

For peer review only - http://bmjopen.bmj.com/site/about/guidelines.xhtml

32. Erchick DJ, Rai B, Agrawal NK, et al. Oral hygiene, prevalence of gingivitis, and associated risk factors among pregnant women in Sarlahi District, Nepal. *BMC oral health* 2019;19(1):2. doi: 10.1186/s12903-018-0681-5 [published Online First: 2019/01/07]
33. Xiong X, Buekens P, Goldenberg RL, et al. Optimal timing of periodontal disease treatment for prevention of adverse pregnancy outcomes: before or during pregnancy? *American journal of obstetrics and gynecology* 2011;205(2):111.e1-6. doi: 10.1016/j.ajog.2011.03.017 [published Online First: 2011/05/31]
34. Yee R, Mishra P. Nepal National Oral Health 'Pathfinder' Survey 2004. Kathmandu, Nepal: Oral Health Focal Point, Ministry of Health, 2004.
35. Kruse AB, Kuerschner AC, Kunze M, et al. Association between high risk for preterm birth and changes in gingiva parameters during pregnancy-a prospective cohort study. *Clinical oral investigations* 2018;22(3):1263-71. doi: 10.1007/s00784-017-2209-9 [published Online First: 2017/10/04]
36. Lopez NJ, Da Silva I, Ipinza J, et al. Periodontal therapy reduces the rate of preterm low birth weight in women with pregnancy-associated gingivitis. *Journal of periodontology* 2005;76(11 Suppl):2144-53. doi: 10.1902/jop.2005.76.11-S.2144 [published Online First: 2005/11/10]
37. Sharma S, Saimbi CS, Koirala B, et al. Effect of various mouthwashes on the levels of interleukin-2 and interferon-gamma in chronic gingivitis. *The Journal of clinical pediatric dentistry* 2008;32(2):111-4. [published Online First: 2008/04/09]
38. Santos A. Evidence-based control of plaque and gingivitis. *Journal of clinical periodontology* 2003;30 Suppl 5:13-6. [published Online First: 2003/06/06]
39. Jeffcoat M, Parry S, Gerlach RW, et al. Use of alcohol-free antimicrobial mouth rinse is associated with decreased incidence of preterm birth in a high-risk population. *American journal of obstetrics and gynecology* 2011;205(4):382.e1-6. doi: 10.1016/j.ajog.2011.07.016 [published Online First: 2011/11/16]
40. Forner L, Larsen T, Kilian M, et al. Incidence of bacteremia after chewing, tooth brushing and scaling in individuals with periodontal inflammation. *Journal of clinical periodontology* 2006;33(6):401-7. doi: 10.1111/j.1600-051X.2006.00924.x [published Online First: 2006/05/09]
41. Madianos PN, Bobetsis YA, Offenbacher S. Adverse pregnancy outcomes (APOs) and periodontal disease: pathogenic mechanisms. *Journal of periodontology* 2013;84(4 Suppl):S170-80. doi: 10.1902/jop.2013.1340015 [published Online First: 2013/05/03]
42. Litonjua LA, Andreana S, Bush PJ, et al. Toothbrushing and gingival recession. *International dental journal* 2003;53(2):67-72. [published Online First: 2003/05/07]
43. Khocht A, Simon G, Person P, et al. Gingival recession in relation to history of hard toothbrush use. *Journal of periodontology* 1993;64(9):900-5. doi: 10.1902/jop.1993.64.9.900 [published Online First: 1993/09/01]
44. Sconyers JR, Crawford JJ, Moriarty JD. Relationship of bacteremia to toothbrushing in patients with periodontitis. *Journal of the American Dental Association (1939)* 1973;87(3):616-22. doi: 10.14219/jada.archive.1973.0453 [published Online First: 1973/09/01]
45. Mobeen N, Jehan I, Banday N, et al. Periodontal disease and adverse birth outcomes: a study from Pakistan. *American journal of obstetrics and gynecology* 2008;198(5):514.e1-8. doi: 10.1016/j.ajog.2008.03.010 [published Online First: 2008/05/06]

For peer review only - http://bmjopen.bmj.com/site/about/guidelines.xhtml

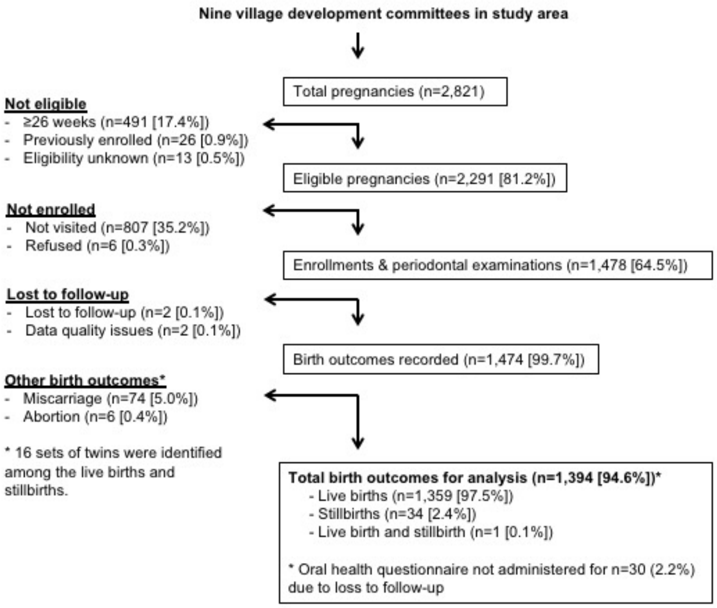

Title: Figure 1: Cohort study participation flow chart

Caption: Data on eligible pregnancies, enrolled pregnancies, refusals, lost to follow-up, and birth outcomes.

254x190mm (72 x 72 DPI)

STROBE Statement—Checklist of items that should be included in reports of ***cohort studies***

	Item No	Recommendation	Page No
**Title and abstract**	1	(*a*) Indicate the study's design with a commonly used term in the title or the abstract<br>(*b*) Provide in the abstract an informative and balanced summary of what was done and what was found	1-3
**Introduction**			
Background/rationale	2	Explain the scientific background and rationale for the investigation being reported	4-5
Objectives	3	State specific objectives, including any prespecified hypotheses	5
**Methods**			
Study design	4	Present key elements of study design early in the paper	6
Setting	5	Describe the setting, locations, and relevant dates, including periods of recruitment, exposure, follow-up, and data collection	6-7
Participants	6	(*a*) Give the eligibility criteria, and the sources and methods of selection of participants. Describe methods of follow-up<br>(*b*) For matched studies, give matching criteria and number of exposed and unexposed	6-9
Variables	7	Clearly define all outcomes, exposures, predictors, potential confounders, and effect modifiers. Give diagnostic criteria, if applicable	7-9
Data sources/ measurement	8*	For each variable of interest, give sources of data and details of methods of assessment (measurement). Describe comparability of assessment methods if there is more than one group	7-9
Bias	9	Describe any efforts to address potential sources of bias	8-9
Study size	10	Explain how the study size was arrived at	8
Quantitative variables	11	Explain how quantitative variables were handled in the analyses. If applicable, describe which groupings were chosen and why	8-9
Statistical methods	12	(*a*) Describe all statistical methods, including those used to control for confounding<br>(*b*) Describe any methods used to examine subgroups and interactions<br>(*c*) Explain how missing data were addressed<br>(*d*) If applicable, explain how loss to follow-up was addressed<br>(*e*) Describe any sensitivity analyses	8-9
**Results**			
Participants	13*	(a) Report numbers of individuals at each stage of study—eg numbers potentially eligible, examined for eligibility, confirmed eligible, included in the study, completing follow-up, and analysed<br>(b) Give reasons for non-participation at each stage<br>(c) Consider use of a flow diagram	10
Descriptive data	14*	(a) Give characteristics of study participants (eg demographic, clinical, social) and information on exposures and potential confounders<br>(b) Indicate number of participants with missing data for each variable of interest<br>(c) Summarise follow-up time (eg, average and total amount)	10-11
Outcome data	15*	Report numbers of outcome events or summary measures over time	10

For peer review only

For peer review only - http://bmjopen.bmj.com/site/about/guidelines.xhtml

Main results	16	(*a*) Give unadjusted estimates and, if applicable, confounder-adjusted estimates and their precision (eg, 95% confidence interval). Make clear which confounders were adjusted for and why they were included<br>(*b*) Report category boundaries when continuous variables were categorized<br>(*c*) If relevant, consider translating estimates of relative risk into absolute risk for a meaningful time period	15-19
Other analyses	17	Report other analyses done—eg analyses of subgroups and interactions, and sensitivity analyses	20-22
**Discussion**			
Key results	18	Summarise key results with reference to study objectives	23
Limitations	19	Discuss limitations of the study, taking into account sources of potential bias or imprecision. Discuss both direction and magnitude of any potential bias	26-27
Interpretation	20	Give a cautious overall interpretation of results considering objectives, limitations, multiplicity of analyses, results from similar studies, and other relevant evidence	27
Generalisability	21	Discuss the generalisability (external validity) of the study results	27
**Other information**			
Funding	22	Give the source of funding and the role of the funders for the present study and, if applicable, for the original study on which the present article is based	28

*Give information separately for exposed and unexposed groups.

Note: An Explanation and Elaboration article discusses each checklist item and gives methodological background and published examples of transparent reporting. The STROBE checklist is best used in conjunction with this article (freely available on the Web sites of PLoS Medicine at http://www.plosmedicine.org/, Annals of Internal Medicine at http://www.annals.org/, and Epidemiology at http://www.epidem.com/). Information on the STROBE Initiative is available at http://www.strobe-statement.org.

For peer review only

For peer review only - http://bmjopen.bmj.com/site/about/guidelines.xhtml

BMJ Open

Risk of preterm birth associated with maternal gingival inflammation and oral hygiene behaviors in rural Nepal: A community-based, prospective cohort study

Journal:	*BMJ Open*
Manuscript ID	bmjopen-2019-036515.R2
Article Type:	Original research
Date Submitted by the Author:	04-Jul-2020
Complete List of Authors:	Erchick, Daniel; Johns Hopkins University Bloomberg School of Public Health, International Health<br>Khatry, Subarna; Nepal Nutrition Intervention Project<br>Agrawal, Nitin; Institute of Medicine, Tribhuhvan University, Department of Dentistry<br>Katz, Joanne; Johns Hopkins University Bloomberg School of Public Health, International Health<br>LeClerq, Steven; Johns Hopkins University Bloomberg School of Public Health, International Health; Nepal Nutrition Intervention Project Sarlahi<br>Rai, Bhola; Nepal Nutrition Intervention Project Sarlahi<br>Reynolds, Mark; University of Maryland School of Dentistry, Department of Advanced Oral Sciences and Therapeutics<br>Mullany, Luke C.; Johns Hopkins University Bloomberg School of Public Health, International Health
**Primary Subject Heading**:	Global health
Secondary Subject Heading:	Epidemiology, Dentistry and oral medicine, Obstetrics and gynaecology, Public health
Keywords:	EPIDEMIOLOGY, OBSTETRICS, ORAL MEDICINE, Community child health < PAEDIATRICS, PERINATOLOGY, PUBLIC HEALTH

SCHOLARONE™
Manuscripts

I, the Submitting Author has the right to grant and does grant on behalf of all authors of the Work (as defined in the below author licence), an exclusive licence and/or a non-exclusive licence for contributions from authors who are: i) UK Crown employees; ii) where BMJ has agreed a CC-BY licence shall apply, and/or iii) in accordance with the terms applicable for US Federal Government officers or employees acting as part of their official duties; on a worldwide, perpetual, irrevocable, royalty-free basis to BMJ Publishing Group Ltd ("BMJ") its licensees and where the relevant Journal is co-owned by BMJ to the co-owners of the Journal, to publish the Work in this journal and any other BMJ products and to exploit all rights, as set out in our licence.

The Submitting Author accepts and understands that any supply made under these terms is made by BMJ to the Submitting Author unless you are acting as an employee on behalf of your employer or a postgraduate student of an affiliated institution which is paying any applicable article publishing charge ("APC") for Open Access articles. Where the Submitting Author wishes to make the Work available on an Open Access basis (and intends to pay the relevant APC), the terms of reuse of such Open Access shall be governed by a Creative Commons licence – details of these licences and which Creative Commons licence will apply to this Work are set out in our licence referred to above.

Other than as permitted in any relevant BMJ Author's Self Archiving Policies, I confirm this Work has not been accepted for publication elsewhere, is not being considered for publication elsewhere and does not duplicate material already published. I confirm all authors consent to publication of this Work and authorise the granting of this licence.

For peer review only

Risk of preterm birth associated with maternal gingival inflammation and oral hygiene behaviors in rural Nepal: A community-based, prospective cohort study

Daniel J. Erchick[a], PhD, MPH, Subarna K. Khatry[b], MBBS, Nitin K. Agrawal[c], MDS, MSc, Joanne Katz[a], ScD, MS, Steven C. LeClerq[a,b], MPH, Bhola Rai[b], Mark A. Reynolds[d], DDS, PhD, MA, Luke C. Mullany[a], PhD, MHS, MS

Affiliations:

[a]Department of International Health, Johns Hopkins Bloomberg School of Public Health, Baltimore, MD, United States

[b]Nepal Nutrition Intervention Project – Sarlahi (NNIPS), Krishna Galli, Lalitpur, Kathmandu, Nepal

[c]Department of Dentistry, Institute of Medicine, Tribhuhvan University. Kathmandu, Nepal

[d]Department of Advanced Oral Sciences and Therapeutics, University of Maryland School of Dentistry, Baltimore, MD, United States

Emails: Khatry SK (skhatry@wlink.com.np), Agrawal NK (cdrnitin@gmail.com), Katz J (jkatz1@jhu.edu), LeClerq SC (sleclerq@mos.com.np), Rai B (bholarai19@gmail.com), Reynolds MA (MReynolds@umaryland.edu), Mullany LC (lmullany@jhu.edu)

Correspondence: Daniel J. Erchick, PhD, MPH. Department of International Health, Johns Hopkins Bloomberg School of Public Health, 615 N. Wolfe Street, Baltimore, MD, 21205, United States. derchick@jhu.edu

Word Count: 3,875

For peer review only

ABSTRACT

Objectives: Observational studies have identified associations between periodontitis and adverse pregnancy outcomes, but randomized controlled trials evaluating the efficacy of periodontal therapy have yielded inconsistent results. Few studies have explored relationships between gingival inflammation and these outcomes or been conducted in rural, low-income communities, where confounding risk factors differ from other settings.

Methods: We conducted a community-based, prospective cohort study with the aim of estimating associations between the extent of gingival inflammation in pregnant women and incidence of preterm birth in rural Nepal. Our primary exposure was gingival inflammation, defined as bleeding on probing (BOP) ≥10%, stratified by BOP <30% and BOP ≥30%. A secondary exposure, mild periodontitis, was defined as ≥2 interproximal sites with probing depth (PD) ≥4 mm (different teeth) or one site with PD≥5 mm. Our primary outcome was preterm birth (<37 weeks gestation). We used Poisson regression to model this relationship, adjusting for potential confounders.

Results: Of 1,394 participants, 554 (39.7%) had gingival inflammation, 54 (3.9%) mild periodontitis, and 197 (14.1%) delivered preterm. In the adjusted regression model, increasing extent of gingival inflammation was associated with a non-significant increase in risk of preterm birth (BOP ≥30% vs. no BOP: aRR 1.37, 95% CI: 0.81, 2.32). A secondary analysis, stratifying participants by when in pregnancy their oral health status was assessed, showed an association between gingival inflammation and preterm birth among women examined in their first trimester

For peer review only - http://bmjopen.bmj.com/site/about/guidelines.xhtml

(BOP ≥30% vs. no BOP: aRR 2.57, 95% CI: 1.11, 5.95), but not later in pregnancy (BOP ≥30% vs. no BOP: aRR 1.05, 95% CI: 0.52, 2.11).

Conclusions: Gingival inflammation in women examined early in pregnancy and poor oral hygiene behaviors were risk factors for preterm birth. Future studies should evaluate community-based oral health interventions that specifically target gingival inflammation, delivered early in or before pregnancy, on preterm birth.

Strengths and Limitations of this Study

- This study collected data through a large, community-based, prospective cohort in a rural, low-resource setting.
- The study population had low prevalence of some established confounders of the relationship of interest, which are common in other populations.
- Some clinical periodontal measures were not collected due to visit time constraints, for example, recession on interproximal sites or gingival index score.
- Preterm birth classification was based upon maternal self-report of last menstrual period (LMP) instead of the gold standard, ultrasound examination.

For peer review only

INTRODUCTION

Annually, 2.5 million babies die prior to 28 days of life, and preterm birth is the leading cause of these deaths.[1] Preterm newborns that survive are at substantial risk of mortality from other causes, long-term disabilities such as neurological and developmental impairments, and non-communicable diseases.[2] In low- and middle-income countries (LMICs), where the majority of preterm births occur, therapeutic interventions are often unavailable and difficult to scale up, especially in communities where many mothers deliver at home or in primary facilities without skilled care (e.g., South Asia).[3]

Periodontal disease includes several inflammatory conditions, typically initiated by oral bacteria, beginning with reversible accumulation of plaque and inflammation of gingival tissue (gingivitis) and progressing to irreversible breakdown of the supportive tissues of the teeth and tooth loss (periodontitis).[4] Onset of new and worsening of existing gingival inflammation during pregnancy are normal and well documented, peaking in the second or third trimester.[5, 6] Major physiological and hormonal changes occur during pregnancy with wide ranging effects on the body, including increased permeability of gingival capillaries, altered immune system activity, and shifts in composition of the sub-gingival microbiome, including proliferation of aggressive bacteria associated with periodontitis, such as *Porphyromonas gingivalis.*[7, 8]

Periodontal disease in pregnant women has been associated with preterm birth and other adverse pregnancy outcomes.[9-11] Yet randomized controlled trials (RCTs) evaluating the impact of periodontal therapy during pregnancy on adverse pregnancy outcomes have produced inconsistent results.[9, 12-18] One meta-analysis found a significant effect of periodontal therapy

For peer review only - http://bmjopen.bmj.com/site/about/guidelines.xhtml

among women at high risk of preterm birth.[19] Although the mechanisms underlying this observed association are unclear, hypotheses include hematogenic translocation of periodontal pathogens or their byproducts to the fetal-placental unit, or action of inflammatory mediators in the periodontium on levels of systemic inflammation.[20]

Alternatively, the observed relationship between periodontal disease in pregnant women and adverse pregnancy outcomes could be the result of unmeasured and uncontrolled confounding factors. Previously described confounders of this relationship, which are commonly controlled for in studies, include age, smoking, multiple birth, previous adverse pregnancy outcomes, ethnicity, and socioeconomic factors.[11] Some studies, however, have proposed the possibility that a genetic inflammatory phenotype could be responsible for increased risk of periodontal disease, or failed periodontal theory, and adverse pregnancy outcomes, particularly spontaneous preterm birth.[21]

Few studies have evaluated whether gingival inflammation is associated with risk of preterm birth or other adverse pregnancy outcomes. Further, studies of this association have been nearly universally facility-based, whether in high- or low-income settings. Understanding this relationship from a population-based perspective in low resource communities can offer certain benefits from an epidemiological perspective. Many populations in LMICs have lower prevalence of important confounding factors of this relationship, such as smoking, alcohol use, and chronic diseases (e.g. hypertension or diabetes). Community-based studies can avoid selection bias associated with hospital-based studies, particularly in populations where home delivery remains common, as is the case in much of South Asia. Given these potential benefits,

For peer review only - http://bmjopen.bmj.com/site/about/guidelines.xhtml

For peer review only

we conducted a community-based, prospective cohort study to estimate the association between gingival inflammation and preterm birth among women in a rural community in the Terai (plains) region of Nepal.

For peer review only

For peer review only - http://bmjopen.bmj.com/site/about/guidelines.xhtml

METHODS

We conducted a community-based, prospective cohort study of maternal gingival inflammation and adverse pregnancy outcomes across nine village development committees (VDCs) in Sarlahi District, Nepal. Eligible participants included married pregnant women between the ages of 15 and 40 who were <26 weeks gestation at the time of enrollment. Participants were identified and determined eligible between January and November 2016 using the infrastructure of a large community-based randomized trial of topical applications for newborn massage, the Nepal Oil Massage Study (NOMS) (NCT01177111), which was actively enrolling a population-based sample of pregnant women in this study area.

Study visits

Study visits were conducted in participant homes because of the wide dispersion of households across this rural community and the impracticality of bringing participants to a central location. Data on participant demographics, vital signs and morbidities during pregnancy, oral hygiene practices, care-seeking, and knowledge, and other characteristics were collected through a series of questionnaires administered over several visits during the course of pregnancy. Data collection teams were notified of the birth outcome by a locally resident study staff member, and the date of birth and other data concerning the mother and newborn were collected as soon as possible after delivery.

Oral health examinations were performed by five auxiliary nurse midwives who were trained for the purpose of this study. Their training lasted 3-4 weeks and included classroom instruction and practice of periodontal techniques conducted by an experienced dentist (NKA) at the Department

For peer review only - http://bmjopen.bmj.com/site/about/guidelines.xhtml

of Dentistry, Institute of Medicine, Tribhuvann University, Kathmandu, Nepal. We estimated the validity of PD measurements of the auxiliary nurse midwives relative to the dentist (NKA), finding percent agreement, weighted kappa scores, and intraclass correlation coefficients, with an allowance of PD ±1 mm, exceeded 99%, 0.7, and 0.9, respectively.[22]

Periodontal measurements

Auxiliary nurse midwives used portable dental equipment to conduct a full mouth examination. Periodontal measurements were made using a color Williams probe (Hu-Friedy, Chicago, IL, USA). PD was measured on six sites per tooth (disto-, mid-, and mesial- aspects of buccal and lingual surfaces) and CEJ-GM distance on two sites per tooth (mid- buccal and lingual aspects), excluding third molars. Presence or absence of BOP was recorded for any buccal or lingual probing site of each tooth. PD values were recorded in millimeters rounded to the next higher whole number. CEJ-GM distances were recorded similarly. If the free gingiva was coronal to the CEJ, the CEJ-GM measurement was recorded as 0. Clinical attachment loss (CAL) was calculated by summing PD and CEJ-GM distances.

Outcome/exposure definitions

Our primary exposure definition, gingival inflammation, was defined according to a classification scheme developed by the American Academy of Periodontology (AAP) and European Federation of Periodontology to update the 1999 classification of periodontal diseases and conditions.[23] Clinical health was defined as all sites PD ≤3 mm and BOP <10%.[24, 25] Gingival inflammation was defined as BOP ≥10%, stratified as localized gingival inflammation (BOP 10%-30%) and generalized gingival inflammation (BOP ≥30%).[24-29]

For peer review only - http://bmjopen.bmj.com/site/about/guidelines.xhtml

We defined secondary exposure definitions for mild and moderate periodontitis based upon a modified version of Centers for Disease Control and AAP updated 2012 case definitions for population-based surveillance of periodontitis.[28, 30] Mild periodontitis was defined as ≥2 interproximal sites with PD≥4 mm (not on the same tooth) or one site with PD≥5 mm. Moderate periodontitis was defined as ≥2 interproximal sites with PD≥5 mm (not on the same tooth). CAL was not included in these definitions as we did not collect this measure at interproximal sites to shorten the visit time; however, clinical attachment level and probing depth are widely considered equivalent measures of periodontitis in younger adults.[31]

Gestational age was calculated using the last menstrual period (LMP) method as recalled by the mother at 5-weekly pregnancy surveillance home visits. Our 5-weekly pregnancy surveillance approach has several benefits, including short recall (≤5 weeks) and pregnancy testing, as compared to traditional LMP approaches, which often rely on LMP recall later in pregnancy or at time of delivery. Preterm birth was defined as a live birth or stillbirth <37 completed weeks of gestation at the time of delivery.

Statistical analysis

Bivariate analyses between participant characteristics and the outcome, preterm birth, were evaluated using t-tests and Poisson regression for continuous and binary/categorical variables, respectively. We calculated unadjusted and adjusted relative risks (RR & aRR) of preterm birth and associated 95% confidence internals (CI) using Poisson regression with robust variance, which was used due to occurrence of convergence issues with other regression methods. Given

For peer review only - http://bmjopen.bmj.com/site/about/guidelines.xhtml

our sample size (n=1,394) and assuming a power of 80%, type I error of 0.05, and our pre-study estimates of population prevalence of preterm birth (17%) and gingival inflammation (40%), we estimated that we could detect a relative risk of preterm birth of roughly >1.5.

Multivariable models were constructed by sequentially adding groups of covariates, beginning with maternal characteristics, oral hygiene behaviors, and socioeconomic factors. Covariates associated with preterm birth at the $p<0.10$ level in bivariate analyses were considered in these regression models. Additional variables, known through previous studies to be confounders of the periodontal disease and preterm birth relationship, were also included in regression models (including age, ethnicity, body mass index (BMI), primiparity, multiple births, and socioeconomic variables (i.e., literacy, education, and indicators of household wealth).

In an effort to remove a possibly attenuating effect of including cases of transient pregnancy-induced gingival inflammation, we examined the relationship stratified by timing of oral exam. Women were categorized into two trimester groups (<13 weeks and ≥13 to <26 weeks gestation). Similar to the primary analysis, for each trimester group we calculated adjusted relative risks (RR & aRR) of preterm birth and associated 95% confidence internals (CI) using Poisson regression with robust variance.

All statistical analyses were performed in STATA 14.2 (StataCorp, College Station, TX, USA). All participants provided written consent for this study. The study received ethical approval from the Institutional Review Board at Johns Hopkins Bloomberg School of Public Health (Baltimore, USA) and the Ethical Review Board of the Nepal Health Research Council (Kathmandu, Nepal).

For peer review only - http://bmjopen.bmj.com/site/about/guidelines.xhtml

Patient and Public Involvement

There was no patient or public involvement in this study.

For peer review only

For peer review only - http://bmjopen.bmj.com/site/about/guidelines.xhtml

RESULTS

Participant characteristics

Between January 11, 2016, and November 26, 2016, among 2,821 pregnancies in the study area, 2,291 (81.2%) participants were identified by the parent trial as eligible for enrollment (<26 weeks gestation) (Figure 1). Among eligible participants, 1,478 (64.5%) were enrolled and six refused participation (0.3%). Another 807 (35.2%) were not visited for the purposes of the study because the eligible participants in the study area initially exceeded the capacity of the auxiliary nurse midwives to enroll women before their gestational age exceeded the exclusion criterion of <26 weeks. For logistical reasons, enrollment of women earlier in pregnancy was prioritized at the start of study enrollment, while enrollment of women later in pregnancy was prioritized towards the end of study enrollment. However, among eligible women, the included and excluded participant groups did not differ by age, education, or socioeconomic factors.

Birth outcomes for the 1,474 women followed until the end of pregnancy were recorded as 1,345 single live births, 14 twin live births, 33 single stillbirths, 1 twin stillbirth, 1 set of twins where 1 was live born and 1 stillborn, 74 miscarriages, and 6 abortions. As our primary outcome was preterm birth, pregnancy was selected as the unit of analysis (and, therefore, twins were counted only once). There were 1,394 total pregnancies for analysis, of which 197 (14.1%) were preterm. Preterm deliveries included 163 (11.7%) moderate preterm (<37 to ≥32 weeks), 27 (1.9%) very preterm (<32 to ≥28 weeks), and 7 (0.5%) extremely preterm (<28 weeks). Over three-quarters of participants (n=1,053, 75.5%) were full term (≥37 weeks) and 144 (10.3%) were post-term (≥42 weeks).

For peer review only - http://bmjopen.bmj.com/site/about/guidelines.xhtml

Baseline demographic, periodontal, oral hygiene, and dental health care seeking characteristics of participants by extent of gingival inflammation were previously reported.[32] At enrollment, mean gestation was 14.5 (SD: 4.3) weeks (range: 6.4 to 27.7 weeks), with 574 women in their first trimester and 820 in their second. Participant age averaged 23.0 ± 4.6 years, and risk of preterm birth was higher (RR 1.93, CI: 1.01, 3.72) for women ≥35 years relative to those 18-35 years (Table 1). Several known confounders of the gingival inflammation and preterm birth relationship had a reported prevalence near 0% in this study population, including smoking and other tobacco use, alcohol use, and hypertension.

For peer review only

Table 1: Participant characteristics at enrollment (n=1,394)

Characteristic	All	Term	Preterm	Mean difference / RR (95% CI)*
**Age**				
Year (mean ± SD)	23.0 ± 4.6	23.0 ± 4.6	23.0 ± 5.1	0.02 (-0.68, 0.72)
**Age (years)**				
<18	151 (10.8)	124 (10.4)	27 (13.7)	1.33 (0.92, 1.93)
18-<35	1216 (87.2)	1053 (88.0)	163 (82.7)	Ref
≥35	27 (1.9)	20 (1.7)	7 (3.6)	**1.93 (1.01, 3.72)**
**Ethnic group**				
Hills (Pahadi)	101 (7.3)	89 (7.4)	12 (6.1)	Ref
Plains (Madeshi)	1292 (92.7)	1107 (92.6)	185 (93.9)	1.21 (0.70, 2.08)
**Height**				
Cm (mean ± SD)	150.8 ± 5.5	150.9 ± 5.6	150.1 ± 5.1	-0.80 (-1.63, 0.03)
**Weight**				
Kg (mean ± SD)	45.8 ± 7.0	46.0 ± 7.1	44.3 ± 5.8	**-1.67 (-2.72, -0.62)**
**BMI**				
Underweight (<18.5 kg)	418 (30.0)	353 (29.5)	65 (33.0)	1.09 (0.83, 1.44)
Normal weight (18.5-<25 kg)	893 (64.1)	766 (64.0)	127 (64.5)	Ref
Overweight or obese (≥25 kg)	83 (6.0)	78 (6.5)	5 (2.5)	0.42, (0.18, 1.01)
**High blood pressure**				
No	1384 (99.3)	1188 (99.2)	196 (99.5)	Ref
Yes	10 (0.7)	9 (0.8)	1 (0.5)	0.71 (0.11, 4.56)
**Gravidity**				
First pregnancy	395 (28.3)	332 (27.7)	63 (32.0)	1.23 (0.92, 1.63)
1-3 previous pregnancies	838 (60.1)	729 (60.9)	109 (55.3)	Ref
≥4 pregnancies	161 (11.5)	136 (11.4)	25 (12.7)	1.19 (0.80, 1.78)
**Urinary or vaginal infection+**				
No	1097 (78.7)	942 (78.7)	155 (78.7)	Ref
Yes	297 (21.3)	255 (21.3)	42 (21.3)	1.00 (0.73, 1.37)

For peer review only - http://bmjopen.bmj.com/site/about/guidelines.xhtml

For peer review only

**Literacy**				
No	751 (53.9)	638 (53.3)	113 (57.4)	Ref
Yes	643 (46.1)	559 (46.7)	84 (42.6)	0.87 (0.67, 1.13)
**Education (years)**				
0	751 (53.9)	634 (53.0)	117 (59.4)	Ref
1-9	386 (27.7)	343 (28.7)	43 (21.8)	**0.72 (0.52, 0.99)**
≥10	256 (18.4)	219 (18.3)	37 (18.8)	0.93 (0.66, 1.31)
**Electricity**				
No	114 (8.2)	97 (8.1)	17 (8.6)	Ref
Yes	1279 (91.8)	1099 (91.9)	180 (91.4)	0.94 (0.60, 1.49)
**House construction material**				
None, thatch, sticks, or bamboo	858 (61.6)	749 (62.6)	109 (55.3)	Ref
Wood planks, bricks, or stone	535 (38.4)	447 (37.4)	88 (44.7)	**1.29 (1.00, 1.68)**
**House roof material**				
None, plastic, thatch, or grass	110 (7.9)	92 (7.7)	18 (9.1)	Ref
Tile/tin/concrete	1283 (92.1)	1104 (92.3)	179 (90.9)	0.85 (0.55, 1.33)
**Latrine**				
No latrine	601 (43.1)	515 (43.1)	86 (43.7)	Ref
Brick, concrete, or pit latrine	792 (56.9)	681 (56.9)	111 (56.3)	0.98 (0.75, 1.27)

Data presented as No. (%) unless otherwise noted

* T-test or relative risk and 95% confidence interval as appropriate

+ Self-reported symptoms of painful urination or foul smelling vaginal discharge during pregnancy

For peer review only

According to our definition, 554 (39.7%) participants had gingival inflammation and 840 (60.3%) were healthy (Table 2). Of those with gingival inflammation, 445 (80.3%) had localized gingival inflammation (BOP <30%) and 109 (19.7%) generalized gingival inflammation (BOP ≥30%). Most participants (n=1,105, 79.3%) had at least one site with BOP. According to our secondary exposure definitions, 54 participants (3.9%) had mild and 4 (0.3%) moderate periodontitis. More participants (n=120, 8.6%) had ≥1 site with PD ≥4 mm. Mean CAL was equivalent to mean PD, with nearly all of CAL ≥4 mm due to pocketing secondary to gingival enlargement, indicating very little recession of the gingiva in this population.

For peer review only

Table 2: Periodontal disease status (n=1,394)

Characteristic	All	Term	Preterm	Rate of preterm
**Health and gingival inflammation**				
Health (All sites PD ≤3 mm and BOP <10%)	840 (60.3)	727 (60.7)	113 (57.4)	13.5%
Gingival inflammation (BOP ≥10% and/or PD ≥4 mm)	554 (39.7)	470 (39.3)	84 (42.6)	15.2%
- Localized gingival inflammation (BOP <30%)	445 (80.3)	379 (80.6)	66 (78.6)	14.8%
- Proportion with no sites PD ≥4 mm	354 (79.6)	299 (78.9)	55 (83.3)	15.5%
- Proportion with ≥1 sites PD ≥4 mm	91 (20.5)	80 (21.2)	11 (16.7)	12.1%
- Generalized gingival inflammation (BOP ≥30%)	109 (19.7)	91 (19.4)	18 (21.4)	16.5%
- Proportion with no sites PD ≥4 mm	80 (73.4)	66 (72.5)	14 (77.8)	17.5%
- Proportion with ≥1 sites PD ≥4 mm	29 (26.6)	25 (27.5)	4 (22.2)	13.8%
**Bleeding on probing (BOP)**				
Percent of sites BOP (mean ± SD)	10.2 ± 12.3	10.1 ± 12.4	10.6 ± 12.0	14.1%*
No sites BOP	289 (20.7)	249 (20.8)	40 (20.3)	13.8%
≥1 site BOP	1,105 (79.3)	949 (79.2)	157 (79.7)	14.2%
≥1 site BOP & BOP <10%	601 (43.1)	521 (43.5)	80 (40.6)	13.3%
BOP ≥10% & BOP <30%	395 (28.3)	336 (28.1)	59 (30.0)	14.9%
BOP ≥30%	109 (7.8)	91 (7.6)	18 (9.1)	16.5%
**Probing depth (PD)**				
Mean PD (mm) (mean ± SD)	1.7 ± 0.3	1.7 ± 0.3	1.7 ± 0.3	14.1%*
Mean PD at direct sites (mm) (mean ± SD)	1.5 ± 0.3	1.5 ± 0.3	1.5 ± 0.2	14.1%*
Percent of sites PD ≥4 mm (mean ± SD)	0.2 ± 1.0	0.2 ± 1.0	0.1 ± 0.9	14.1%*
≥1 site PD ≥4 mm	120 (8.6)	105 (8.8)	15 (7.6)	12.5%
**Clinical attachment loss (CAL)**				
Mean CAL at direct sites (mm) (mean ± SD)	1.7 ± 0.3	1.7 ± 0.3	1.7 ± 0.3	14.1%*
≥1 site recession ≥1 mm	173 (12.4)	154 (12.9)	19 (9.6)	11.0%
≥1 site CAL ≥4 mm	196 (14.1)	172 (14.4)	24 (12.2)	12.2%
Percent of CAL ≥4 mm due to pocketing (mean ± SD)	99.6 ± 1.5	99.6 ± 1.5	99.8 ± 0.9	14.1%*
Percent of CAL ≥4 mm due to recession (mean ± SD)	0.4 ± 1.5	0.4 ± 1.5	0.2 ± 0.9	14.1%*

Data presented as No. (%) unless otherwise noted

* Preterm birth rate among all participants

For peer review only - http://bmjopen.bmj.com/site/about/guidelines.xhtml

Several poor oral hygiene behaviors were associated with increased risk of preterm birth, including extended teeth cleaning time (≥30 mins vs. <5 mins: RR 2.48, 95% CI: 1.41, 4.39), use of a non-traditional dentifrice (i.e., sand, ash, oil, or gul) (RR 2.22, 95% CI: 1.44, 3.42). Teeth cleaning episodes per week (≥14 vs. <7 times: RR 0.55, 95% CI: 0.29, 1.02) was associated with decreased risk of preterm birth. Risk of preterm birth increased with a dose response pattern for women who practiced multiple poor oral hygiene behaviors, defined as extended cleaning time per episode (≥5 minutes), infrequent cleaning episodes (<7 times per week), and use of a traditional dentifrice. Incidence of preterm birth was 17/208 (8.2%) for women with no poor behaviors, 155/1,098 (14.1%) for women with one, and 25/88 (28.4%) for those with two.

Primary analysis: Association between maternal gingival inflammation and preterm birth

Incidence of preterm birth by periodontal status was 113/840 (13.5%) for health and 84/554 (15.2%) for gingival inflammation. Incidence of preterm birth increased from 120/890 (13.5%) for women with either health (no sites BOP) or BOP <10% to 59/395 (14.9%) for women with localized gingival inflammation (BOP ≥10% & <30%) to 18/109 (16.5%) for women with generalized gingival inflammation (BOP ≥30%).

Our binary definition of gingival inflammation (BOP ≥10%) was not associated with preterm birth in the crude model (RR 1.13, 95% CI: 0.87, 1.46) or after adjusting for confounders (aRR 1.16, 95% CI: 0.90, 1.51). In the final adjusted model, increasing extent of gingival inflammation was associated with a non-significant increase in risk of preterm birth (BOP ≥30% vs. no BOP: aRR 1.37, 95% CI: 0.81, 2.32) (Table 3). In this model, several oral hygiene behaviors were associated with preterm birth. Extended cleaning time (≥30 mins vs. <5 mins: aRR 2.44, 95% CI:

For peer review only - http://bmjopen.bmj.com/site/about/guidelines.xhtml

1.34, 4.43), use of a traditional dentifrice (n=53, 3.9%) (aRR 2.17, 95% CI: 1.39, 3.38), and fewer teeth cleaning episodes per week (≥14 times vs. 1-6 times: aRR 0.46, 95% CI: 0.25, 0.84) were associated with increased risk of preterm birth.

For peer review only

For peer review only - http://bmjopen.bmj.com/site/about/guidelines.xhtml

Table 3: Adjusted relative risks (aRR) between maternal gingival inflammation and preterm birth

		Preterm birth aRR (95% CI)		
**Characteristic**	**Unadjusted model (n=1,394)**	**Model 1 (n=1,393)**	**Model 2 (n=1,358)**	**Final model (n=1,357)**
**Gingival inflammation**				
No sites BOP	Ref	Ref	Ref	Ref
≥1 site BOP & BOP <10%	0.96 (0.68, 1.37)	0.99 (0.70, 1.40)	1.08 (0.76, 1.54)	1.07 (0.75, 1.51)
BOP ≥10% & BOP <30%	1.08 (0.74, 1.57)	1.11 (0.77, 1.61)	1.19 (0.82, 1.73)	1.17 (0.80, 1.69)
BOP ≥30%	1.19 (0.72, 1.99)	1.24 (0.74, 2.08)	1.34 (0.79, 2.28)	1.37 (0.81, 2.32)
**Age (years)**				
<18		1.21 (0.80, 1.85)	1.17 (0.77, 1.76)	1.21 (0.80, 1.84)
18-<35		Ref	Ref	Ref
≥35		**2.17 (1.14, 4.11)**	1.82 (0.95, 3.49)	1.65 (0.88, 3.10)
**Pahadi/Madeshi**				
Pahadi		Ref	Ref	Ref
Madeshi		1.13 (0.65, 1.97)	0.95 (0.55, 1.66)	0.97 (0.55, 1.70)
**BMI (kg)**				
Underweight (<18.5)		1.10 (0.83, 1.45)	1.11 (0.85, 1.47)	1.15 (0.87, 1.52)
Normal weight (18.5-<25)		Ref	Ref	Ref
Overweight or obese (≥25)		0.45 (0.18, 1.08)	0.49 (0.20, 1.18)	0.47 (0.20, 1.13)
**Primiparous**				
No		Ref	Ref	Ref
Yes		1.17 (0.86, 1.60)	1.25 (0.92, 1.71)	1.26 (0.91, 1.75)
**Multiple births**				
No		Ref	Ref	Ref
Yes		**4.46 (2.88, 6.92)**	**3.76 (2.46, 5.75)**	**3.38 (2.09, 5.46)**
**Teeth cleaning time per episode (mins)**				
<5			Ref	Ref
5-<30			**1.77 (1.07, 2.94)**	**1.83 (1.11, 3.04)**
≥30			**2.37 (1.31, 4.27)**	**2.44 (1.34, 4.43)**
**Teeth cleaning episodes per week**				
1-6			Ref	Ref

For peer review only - http://bmjopen.bmj.com/site/about/guidelines.xhtml
For peer review only

7-13	0.64 (0.39, 1.05)	**0.62 (0.39, 1.00)**
≥14	**0.48 (0.26, 0.89)**	**0.46 (0.25, 0.84)**
**Use of traditional dentifrice**		
No	Ref	Ref
Yes	**2.13 (1.37, 3.31)**	**2.17 (1.39, 3.38)**
**Literacy**		
No		Ref
Yes		1.51 (0.85, 2.66)
**Education (years)**		
0		Ref
1-9		**0.50 (0.27, 0.92)**
≥10		0.65 (0.35, 1.21)
**House construction material**		
None, plastic, thatch, or grass		Ref
Wood planks, bricks, or stone		**1.47 (1.11, 1.93)**
**Roof construction material**		
None, plastic, thatch, or grass		Ref
Tin, tile, or concrete		0.85 (0.55, 1.33)

For peer review only

Secondary analysis: Stratification by gestational age at periodontal examination

In a secondary analysis, participants were stratified by their trimester at the time of the periodontal examination (1st trimester: n=574; 2nd trimester: n=820). Among women with dental exams in the first trimester, incidence of preterm birth increased from 47/381 (12.3%) for women with either health (no sites BOP) or BOP <10% to 28/158 (17.7%) for women with localized gingival inflammation (BOP ≥10% & <30%) to 8/35 (22.9%) for women with generalized gingival inflammation (BOP ≥30%). Our binary definition of gingival inflammation (BOP ≥10%) in the first trimester was associated with preterm (aRR 1.62, 95% CI: 1.08, 2.42), but not in the second trimester (aRR 0.92, 95% CI: 0.65, 1.30), after adjusting for confounders. In the final adjusted model stratified by trimester, there was a positive relationship between gingival inflammation and risk of preterm birth among women in the first trimester (BOP ≥30% vs. no BOP: aRR 2.57, 95% CI: 1.11, 5.95), but not among women in their second trimester (BOP ≥30% vs. no BOP: aRR 1.05, 95% CI: 0.52, 2.11) (Table 4).

Our secondary exposure definition for mild periodontitis was not associated with preterm in a binary comparison or the crude or adjusted regression models for our primary or secondary analyses. These analyses were not conducted for moderate periodontitis given the low prevalence of the condition in this study population.

For peer review only - http://bmjopen.bmj.com/site/about/guidelines.xhtml

Table 4: Adjusted relative risks (aRR) between maternal gingival inflammation and preterm birth stratified by trimester at periodontal examination visit

	Preterm birth aRR (95% CI)	
**Characteristic**	**Women in 1st trimester (n=559)**	**Women in 2nd trimester (n=798)**
**Gingival inflammation**		
No sites BOP	Ref	Ref
≥1 site BOP & BOP <10%	1.51 (0.81, 2.84)	0.93 (0.60, 1.44)
BOP ≥10% & BOP <30%	**1.91 (1.00, 3.63)**	0.88 (0.55, 1.42)
BOP ≥30%	**2.57 (1.11, 5.95)**	1.05 (0.52, 2.11)
**Age (years)**		
<18	1.06 (0.52, 2.18)	1.42 (0.85, 2.36)
18-<35	Ref	Ref
≥35	0.97 (0.28, 3.38)	**2.42 (1.14, 5.14)**
**Pahadi/Madeshi**		
Pahadi	Ref	Ref
Madeshi	1.15 (0.46, 2.90)	0.82 (0.42, 1.60)
**BMI (kg)**		
Underweight (<18.5)	1.44 (0.95, 2.18)	0.96 (0.65, 1.42)
Normal weight (18.5-<25)	Ref	Ref
Overweight or obese (≥25)	0.72 (0.19, 2.78)	0.33 (0.11, 1.06)
**Primiparous**		
No	Ref	Ref
Yes	1.41 (0.84, 2.34)	1.13 (0.74, 1.72)
**Multiple births**		
No	Ref	Ref
Yes	2.57 (0.92, 7.15)	**4.38 (2.57, 7.47)**
**Teeth cleaning time per episode (mins)**		
<5	Ref	Ref
5-<30	1.40 (0.71, 2.75)	**2.42 (1.13, 5.19)**
≥30	2.06 (0.92, 4.57)	**2.93 (1.19, 7.23)**
**Teeth cleaning episodes per week**		
1-6	Ref	Ref
7-13	0.87 (0.39, 1.93)	**0.49 (0.28, 0.85)**
≥14	0.49 (0.18, 1.31)	**0.46 (0.22, 0.95)**
**Use of traditional dentifrice**		
No	Ref	Ref
Yes	1.61 (0.63, 4.10)	**2.58 (1.56, 4.26)**
**Literacy**		
No	Ref	Ref
Yes	1.97 (0.78, 4.96)	1.25 (0.60, 2.61)
**Education (years)**		
0	Ref	Ref
1-9	**0.34 (0.13, 0.89)**	0.67 (0.31, 1.45)

For peer review only - http://bmjopen.bmj.com/site/about/guidelines.xhtml

≥10	0.41 (0.15, 1.11)	0.93 (0.41, 2.10)
**House construction material**		
None, plastic, thatch, or grass	Ref	Ref
Wood planks, bricks, or stone	1.33 (0.84, 2.11)	**1.57 (1.09, 2.25)**
**Roof construction material**		
None, plastic, thatch, or grass	Ref	Ref
Tin, tile, or concrete	0.80 (0.39, 1.65)	0.90 (0.51, 1.59)

For peer review only

DISCUSSION

In our community-based, prospective cohort study, gingival inflammation was an independent risk factor for preterm birth among women examined during their first, but not second, trimester, after adjusting for potential confounding risk factors. Our finding suggests that gingival infection and inflammatory burden early in pregnancy, potentially originating prior to conception, could be responsible for the observed relationship, which becomes obscured in the second trimester by the presence of women with initially healthy periodontal conditions who develop pregnancy-induced gingival inflammation. This result is consistent with studies that have reported that active, and especially progressive, periodontal infection is most harmful early in pregnancy.[13] This also supports the hypothesis, proposed to explain the mixed results of RCTs of this association, that intervening on women with periodontal disease later in pregnancy, or even after conception, may be too late to affect the proposed causal pathway.[33]

Most previous studies have considered the relationship between varying definitions and severities of periodontitis and preterm birth. A meta-analysis of seven cohort studies estimated a pooled relative risk of preterm of 1.70 (1.03, 2.81) for pregnant women with periodontitis versus health.[10] Prevalence of periodontitis in our study population was low, although not dissimilar to previous surveys of periodontal conditions among this age group in Nepal.[34] The low prevalence of periodontitis in this population is likely attributable to the population's low age (median 22.2, IQR: 19.7, 25.5; mean 23.0, SD: 4.6) and absence of other common risk factors for the condition. An unpublished review of community-based studies of the periodontal disease and preterm birth relationship in low- and middle-income countries conducted by our research team found mean maternal ages ranging from 26 to 29. A previous sub-analysis, which was nested within this cohort and conducted by our research team, assessed various potential risk factors for

For peer review only - http://bmjopen.bmj.com/site/about/guidelines.xhtml

gingivitis in pregnant women in this population, identifying maternal age, maternal short stature, Pahadi ethnicity (vs. Madeshi), and some indicators of low socioeconomic status and poor oral hygiene behaviors as significant.[32] Unlike the majority of studies of this association, particularly in high-income countries, the prevalence of important confounders, such as smoking, alcohol use, and morbidities associated with chronic disease, were nearly absent in our study population. An association between periodontitis and preterm birth may have been seen in our population had there been a higher prevalence and severity of periodontitis, as would be expected in an older population.

Few observational or interventional studies have considered gingival inflammation as the primary exposure definition when investigating the relationship between periodontal disease and preterm birth. A cohort study by Kruse et al. (2008) found an association between gingivitis and high risk of preterm birth among women without periodontitis in a hospital setting in Germany.[35] A trial by Lopez et al. (2005) of women with gingivitis in a hospital setting in Santiago, Chile, demonstrated a significantly higher risk of preterm low birth weight among women with gingivitis who received periodontal treatment after delivery compared to those that received treatment during pregnancy (<28 weeks) (aOR: 2.76, 95% CI: 1.29, 5.88).[36] Gingival inflammation requires less intensive treatment than periodontitis, and can include improved oral hygiene, use of an antiseptic oral rinse, and periodontal therapy, interventions that could be feasibly delivered in a rural community setting. While antiseptic oral rinses are known for their ability to reduce plaque and control inflammation, their action on pregnancy outcomes is underexplored.[37, 38] However, Jeffcoat et al. (2011) found a reduction in preterm birth in a high-risk population with the use of cetylpyridinium chloride oral rinse intervention.[39]

For peer review only - http://bmjopen.bmj.com/site/about/guidelines.xhtml

Extent and severity of gingival inflammation, independent of traditional clinical measures of periodontitis, such as CAL or PD, have been associated with magnitude of bacteremia.[40] Transient bacteremia, facilitated by ulceration, inflammation, and the increased vascular permeability of the gingiva, could pose increased risk for adverse pregnancy outcomes.[41] In this case, treatment of traditional clinical measures of periodontitis – without reducing infection, inflammation, and, potentially, the presence of specific harmful pathogens – could fail to disrupt the proposed causal pathway between exposure and outcome. Further, if such bacteremia were to occur early in pregnancy, treatment of clinical conditions later in pregnancy would be too late to eliminate exposure of the placenta and/or fetus to these pathogens, and hence would not decrease of risk of adverse pregnancy outcomes.[41]

Poor oral hygiene behaviors, including extended teeth cleaning time (≥5 mins), infrequent teeth cleaning (<7 times per week), and use of traditional dentifrice (i.e., sand, ash, oil, or gul) or datiwan, were also associated with increased risk of preterm birth in our analysis. Studies have shown that the mechanical manipulation involved in brushing, particularly frequent or forceful brushing or use of brushes with hard filaments, can lead to gingival abrasion, recession, and bacteremia.[42-44] Some have posited that bacteremia caused by mechanical manipulation of the gingiva involved in periodontal therapy may increase risk of adverse pregnancy outcomes, attenuating observation of any true effect in interventional studies of this association.[33] More research is needed to understand the extent to which poor oral hygiene behaviors in pregnant women, including common traditional practices, such as datiwan use, could – via local

For peer review only - http://bmjopen.bmj.com/site/about/guidelines.xhtml

inflammatory responses or increased access of periodontal bacteria or their byproducts to the bloodstream – initiate a cascade of effects that ultimately influences the pregnancy outcome.

An important strength of our study was that we utilized a population-based sample, compared to previous observational studies of this relationship, which were primarily conducted in health facility settings, introducing risk of selection bias associated with the likelihood of women in the target population delivering in a particular facility. The potential for selection bias to influence the measure of association in a facility-based study is especially high in populations where a substantial proportion of women deliver at home, as is the case in this area of rural Nepal, where roughly half of women deliver in a facility. In the unpublished review conducted by our research team, we found one study of this association, by Mobeen et al. (2008), that used a population-based sample.[45] Conducted in a periurban area of Hyderabad, Pakistan, the authors reported significant associations of varying degrees between measures of periodontal disease and neonatal death, perinatal death, and stillbirth.[45]

A limitation of this study was the collection of clinical recession measures from only the direct buccal and lingual surfaces. In the absence of these data, we likely underestimated the burden of periodontal disease among pregnant women in this study, potentially attenuating our measure of association. We were unable to control for some confounders of this relationship, including previous preterm birth, certain chronic diseases (e.g., diabetes), and we only used a proxy (self-reported symptoms) for urinary tract and vaginal infections. Conducting periodontal exams in participant homes, where light and other conditions are variable, may have introduced a level of measurement error in our exposure that could not be quantified or controlled for given the setting

For peer review only - http://bmjopen.bmj.com/site/about/guidelines.xhtml

and logistical constraints of our study. Lastly, preterm birth was based upon maternal self-report of last menstrual period (LMP) instead of ultrasound examination. If our LMP estimates were less accurate among women examined later in pregnancy, due to longer maternal recall relative to women examined earlier in pregnancy, this could have resulted in non-differential misclassification of our binary outcome, preterm birth, which would tend to attenuate observation of a true relationship among women examined in the second trimester.

CONCLUSION

Our study identified gingival inflammation as an independent risk factor for preterm birth among women examined early in pregnancy. Women non-compliant with oral hygiene care recommendations were at substantially increased risk of preterm birth. These findings highlight the importance of adherence to proper home oral self-care in pregnant women and women expecting to become pregnant. Oral health policies and education programs should include tailored approaches to encourage improved self-care practices in this important population. Future studies should evaluate the effectiveness of community-based oral health interventions that specifically target gingival inflammation – delivered to women early in or prior to pregnancy – on the incidence of preterm birth and other adverse pregnancy outcomes in low-income countries with high risk for these outcomes.

For peer review only

For peer review only - http://bmjopen.bmj.com/site/about/guidelines.xhtml

Acknowledgements:
The authors thank the auxiliary nurse midwives who conducted the periodontal examinations: Kaushila Chaudhary, Lalita Lama, Gayatri Mahat, Shanti Sharma, and Santoshi Kumari. Thank you to Roshan Chaudhary for serving as the laboratory technician. We also wish a special thank you to the women from Sarlahi District, Nepal, for participating in this study.

This study was carried out in close collaboration with our implementing partner, Nepal Netra Jyoti Sangh, under the auspices of the Social Welfare Council of the Government of Nepal.

Competing interests: The authors declare that they have no competing interests.

Funding source: This work was supported by the National Institute for Child Health and Development [HD060712] and the Bill & Melinda Gates Foundation [OPP1084399, OPP1131701].

Trial registration: ClinicalTrials.gov Identifier: NCT01177111 (Nepal Oil Massage Study) and NCT02788786 (Pilot Trial).

Patients or public involvement: Patients or the public were not involved in the design, or conduct, or reporting, or dissemination plans of our research.

Data availability: All data files, codebooks, and related manuscripts are available from the JHU Data Archive (https://doi.org/10.7281/T1/ZPGBJW).

Abbreviations:

Adjusted relative risk (aRR)

Bleeding on probing (BOP)

Body mass index (BMI)

Cemento-enamel junction (CEJ)

Clinical attachment loss (CAL)

Confidence intervals (CI)

Gingival margin (GM)

For peer review only

Nepal Oil Massage Study (NOMS)

Relative risk (RR)

Probing depth (PD)

Standard deviation (SD)

Authors' contribution:

Dr. Erchick conceptualized and designed the study, developed field implementation protocols, led data collection in the field, conducted the analysis, and wrote the manuscript.

Dr. Khatry conceptualized and designed the study, oversaw field implementation and ensured quality data collection, and provided comments on the manuscript.

Dr. Agrawal conceptualized and designed the study, trained and oversaw the data collectors, and provided comments on the manuscript.

Dr. Katz conceptualized and designed the study, ensured quality data collection, advised on analytic approach, and provided comments on the manuscript.

Mr. LeClerq conceptualized and designed the study, supported overall implementation in the field, and provided comments on the manuscript.

Mr. Rai contributed to field implementation, ensured quality control of field procedures, and provided comments on the manuscript.

Dr. Reynolds conceptualized and designed the study, contributed to the analysis and interpretation of the results, and provided comments on the manuscript.

For peer review only - http://bmjopen.bmj.com/site/about/guidelines.xhtml

Dr. Mullany conceptualized and designed the study, obtained funding for the study, oversaw implementation of data collection, obtained ethical approvals, advised on analytic approach, and provided edits and comments on the manuscript.

All authors approved the final manuscript as submitted and agree to be accountable for all aspects of the work.

For peer review only

For peer review only - http://bmjopen.bmj.com/site/about/guidelines.xhtml

REFERENCES

1. United Nations Inter-agency Group for Child Mortality Estimation (UN IGME). Levels & Trends in Child Mortality. In: UNICEF, ed. New York, 2018.
2. Blencowe H, Cousens S, Chou D, et al. Born too soon: the global epidemiology of 15 million preterm births. *Reproductive health* 2013;10 Suppl 1:S2. doi: 10.1186/1742-4755-10-s1-s2 [published Online First: 2014/03/15]
3. Barros FC, Bhutta ZA, Batra M, et al. Global report on preterm birth and stillbirth (3 of 7): evidence for effectiveness of interventions. *BMC pregnancy and childbirth* 2010;10 Suppl 1:S3. doi: 10.1186/1471-2393-10-s1-s3 [published Online First: 2010/03/27]
4. Brown LJ, Loe H. Prevalence, extent, severity and progression of periodontal disease. *Periodontology 2000* 1993;2:57-71. [published Online First: 1993/06/01]
5. Loe H, Silness J. Periodontal disease in pregnancy. I. Prevalence and severity. *Acta odontologica Scandinavica* 1963;21:533-51. [published Online First: 1963/12/01]
6. Laine MA. Effect of pregnancy on periodontal and dental health. *Acta odontologica Scandinavica* 2002;60(5):257-64. [published Online First: 2002/11/07]
7. Figuero E, Carrillo-de-Albornoz A, Martin C, et al. Effect of pregnancy on gingival inflammation in systemically healthy women: a systematic review. *Journal of clinical periodontology* 2013;40(5):457-73. doi: 10.1111/jcpe.12053 [published Online First: 2013/04/06]
8. Wu M, Chen SW, Jiang SY. Relationship between gingival inflammation and pregnancy. *Mediators of inflammation* 2015;2015:623427. doi: 10.1155/2015/623427 [published Online First: 2015/04/16]
9. Offenbacher S, Katz V, Fertik G, et al. Periodontal infection as a possible risk factor for preterm low birth weight. *Journal of periodontology* 1996;67(10 Suppl):1103-13. doi: 10.1902/jop.1996.67.10s.1103 [published Online First: 1996/10/01]
10. Chambrone L, Guglielmetti MR, Pannuti CM, et al. Evidence grade associating periodontitis to preterm birth and/or low birth weight: I. A systematic review of prospective cohort studies. *Journal of clinical periodontology* 2011;38(9):795-808. doi: 10.1111/j.1600-051X.2011.01755.x [published Online First: 2011/06/29]
11. Ide M, Papapanou PN. Epidemiology of association between maternal periodontal disease and adverse pregnancy outcomes--systematic review. *Journal of periodontology* 2013;84(4 Suppl):S181-94. doi: 10.1902/jop.2013.134009 [published Online First: 2013/05/03]
12. Offenbacher S, Lieff S, Boggess KA, et al. Maternal periodontitis and prematurity. Part I: Obstetric outcome of prematurity and growth restriction. *Annals of periodontology / the American Academy of Periodontology* 2001;6(1):164-74. doi: 10.1902/annals.2001.6.1.164 [published Online First: 2002/03/13]
13. Offenbacher S, Boggess KA, Murtha AP, et al. Progressive periodontal disease and risk of very preterm delivery. *Obstetrics and gynecology* 2006;107(1):29-36. doi: 10.1097/01.aog.0000190212.87012.96 [published Online First: 2006/01/06]
14. Jeffcoat MK, Geurs NC, Reddy MS, et al. Periodontal infection and preterm birth: results of a prospective study. *Journal of the American Dental Association (1939)* 2001;132(7):875-80. [published Online First: 2001/08/02]
15. Michalowicz BS, Hodges JS, DiAngelis AJ, et al. Treatment of periodontal disease and the risk of preterm birth. *The New England journal of medicine* 2006;355(18):1885-94. doi: 10.1056/NEJMoa062249 [published Online First: 2006/11/03]
16. Offenbacher S, Beck JD, Jared HL, et al. Effects of periodontal therapy on rate of preterm delivery: a randomized controlled trial. *Obstetrics and gynecology* 2009;114(3):551-9. doi: 10.1097/AOG.0b013e3181b1341f [published Online First: 2009/08/25]

For peer review only - http://bmjopen.bmj.com/site/about/guidelines.xhtml

17. Offenbacher S, Lin D, Strauss R, et al. Effects of periodontal therapy during pregnancy on periodontal status, biologic parameters, and pregnancy outcomes: a pilot study. *Journal of periodontology* 2006;77(12):2011-24. doi: 10.1902/jop.2006.060047 [published Online First: 2007/01/11]
18. Iheozor-Ejiofor Z, Middleton P, Esposito M, et al. Treating periodontal disease for preventing adverse birth outcomes in pregnant women. *The Cochrane database of systematic reviews* 2017;6:Cd005297. doi: 10.1002/14651858.CD005297.pub3 [published Online First: 2017/06/13]
19. Kim AJ, Lo AJ, Pullin DA, et al. Scaling and root planing treatment for periodontitis to reduce preterm birth and low birth weight: a systematic review and meta-analysis of randomized controlled trials. *Journal of periodontology* 2012;83(12):1508-19. doi: 10.1902/jop.2012.110636 [published Online First: 2012/03/02]
20. Cetin I, Pileri P, Villa A, et al. Pathogenic mechanisms linking periodontal diseases with adverse pregnancy outcomes. *Reproductive sciences (Thousand Oaks, Calif)* 2012;19(6):633-41. doi: 10.1177/1933719111432871 [published Online First: 2012/03/17]
21. Jeffcoat MK, Jeffcoat RL, Tanna N, et al. Association of a common genetic factor, PTGER3, with outcome of periodontal therapy and preterm birth. *Journal of periodontology* 2014;85(3):446-54. doi: 10.1902/jop.2013.130006 [published Online First: 2013/06/29]
22. Erchick DJ, Agrawal NK, Khatry SK, et al. Feasibility of training community health workers to conduct periodontal examinations: a validation study in rural Nepal. *BMC Health Services Research* 2020;20(1):412. doi: 10.1186/s12913-020-05276-5
23. Armitage GC. Development of a classification system for periodontal diseases and conditions. *Annals of periodontology / the American Academy of Periodontology* 1999;4(1):1-6. doi: 10.1902/annals.1999.4.1.1 [published Online First: 2000/06/23]
24. Chapple ILC, Mealey BL, Van Dyke TE, et al. Periodontal health and gingival diseases and conditions on an intact and a reduced periodontium: Consensus report of workgroup 1 of the 2017 World Workshop on the Classification of Periodontal and Peri-Implant Diseases and Conditions. *Journal of periodontology* 2018;89 Suppl 1:S74-s84. doi: 10.1002/jper.17-0719 [published Online First: 2018/06/22]
25. Trombelli L, Farina R, Silva CO, et al. Plaque-induced gingivitis: Case definition and diagnostic considerations. *Journal of periodontology* 2018;89 Suppl 1:S46-s73. doi: 10.1002/jper.17-0576 [published Online First: 2018/06/22]
26. Holtfreter B, Albandar JM, Dietrich T, et al. Standards for reporting chronic periodontitis prevalence and severity in epidemiologic studies: Proposed standards from the Joint EU/USA Periodontal Epidemiology Working Group. *Journal of clinical periodontology* 2015;42(5):407-12. doi: 10.1111/jcpe.12392 [published Online First: 2015/03/27]
27. Papapanou PN, Sanz M, Buduneli N, et al. Periodontitis: Consensus report of workgroup 2 of the 2017 World Workshop on the Classification of Periodontal and Peri-Implant Diseases and Conditions. *Journal of periodontology* 2018;89 Suppl 1:S173-s82. doi: 10.1002/jper.17-0721 [published Online First: 2018/06/22]
28. Tonetti MS, Greenwell H, Kornman KS. Staging and grading of periodontitis: Framework and proposal of a new classification and case definition. *Journal of periodontology* 2018;89 Suppl 1:S159-s72. doi: 10.1002/jper.18-0006 [published Online First: 2018/06/22]
29. World Health Organization. Epidemiology, etiology, and prevention of periodontal diseases. *World Health Organ Tech Rep Ser* 1978 [published Online First: 1978/01/01]
30. Eke PI, Page RC, Wei L, et al. Update of the case definitions for population-based surveillance of periodontitis. *Journal of periodontology* 2012;83(12):1449-54. doi: 10.1902/jop.2012.110664 [published Online First: 2012/03/17]

For peer review only - http://bmjopen.bmj.com/site/about/guidelines.xhtml

31. Page RC, Eke PI. Case definitions for use in population-based surveillance of periodontitis. *Journal of periodontology* 2007;78(7 Suppl):1387-99. doi: 10.1902/jop.2007.060264 [published Online First: 2007/08/19]
32. Erchick DJ, Rai B, Agrawal NK, et al. Oral hygiene, prevalence of gingivitis, and associated risk factors among pregnant women in Sarlahi District, Nepal. *BMC oral health* 2019;19(1):2. doi: 10.1186/s12903-018-0681-5 [published Online First: 2019/01/07]
33. Xiong X, Buekens P, Goldenberg RL, et al. Optimal timing of periodontal disease treatment for prevention of adverse pregnancy outcomes: before or during pregnancy? *American journal of obstetrics and gynecology* 2011;205(2):111.e1-6. doi: 10.1016/j.ajog.2011.03.017 [published Online First: 2011/05/31]
34. Yee R, Mishra P. Nepal National Oral Health 'Pathfinder' Survey 2004. Kathmandu, Nepal: Oral Health Focal Point, Ministry of Health, 2004.
35. Kruse AB, Kuerschner AC, Kunze M, et al. Association between high risk for preterm birth and changes in gingiva parameters during pregnancy-a prospective cohort study. *Clinical oral investigations* 2018;22(3):1263-71. doi: 10.1007/s00784-017-2209-9 [published Online First: 2017/10/04]
36. Lopez NJ, Da Silva I, Ipinza J, et al. Periodontal therapy reduces the rate of preterm low birth weight in women with pregnancy-associated gingivitis. *Journal of periodontology* 2005;76(11 Suppl):2144-53. doi: 10.1902/jop.2005.76.11-S.2144 [published Online First: 2005/11/10]
37. Sharma S, Saimbi CS, Koirala B, et al. Effect of various mouthwashes on the levels of interleukin-2 and interferon-gamma in chronic gingivitis. *The Journal of clinical pediatric dentistry* 2008;32(2):111-4. [published Online First: 2008/04/09]
38. Santos A. Evidence-based control of plaque and gingivitis. *Journal of clinical periodontology* 2003;30 Suppl 5:13-6. [published Online First: 2003/06/06]
39. Jeffcoat M, Parry S, Gerlach RW, et al. Use of alcohol-free antimicrobial mouth rinse is associated with decreased incidence of preterm birth in a high-risk population. *American journal of obstetrics and gynecology* 2011;205(4):382.e1-6. doi: 10.1016/j.ajog.2011.07.016 [published Online First: 2011/11/16]
40. Forner L, Larsen T, Kilian M, et al. Incidence of bacteremia after chewing, tooth brushing and scaling in individuals with periodontal inflammation. *Journal of clinical periodontology* 2006;33(6):401-7. doi: 10.1111/j.1600-051X.2006.00924.x [published Online First: 2006/05/09]
41. Madianos PN, Bobetsis YA, Offenbacher S. Adverse pregnancy outcomes (APOs) and periodontal disease: pathogenic mechanisms. *Journal of periodontology* 2013;84(4 Suppl):S170-80. doi: 10.1902/jop.2013.1340015 [published Online First: 2013/05/03]
42. Litonjua LA, Andreana S, Bush PJ, et al. Toothbrushing and gingival recession. *International dental journal* 2003;53(2):67-72. [published Online First: 2003/05/07]
43. Khocht A, Simon G, Person P, et al. Gingival recession in relation to history of hard toothbrush use. *Journal of periodontology* 1993;64(9):900-5. doi: 10.1902/jop.1993.64.9.900 [published Online First: 1993/09/01]
44. Sconyers JR, Crawford JJ, Moriarty JD. Relationship of bacteremia to toothbrushing in patients with periodontitis. *Journal of the American Dental Association (1939)* 1973;87(3):616-22. doi: 10.14219/jada.archive.1973.0453 [published Online First: 1973/09/01]
45. Mobeen N, Jehan I, Banday N, et al. Periodontal disease and adverse birth outcomes: a study from Pakistan. *American journal of obstetrics and gynecology* 2008;198(5):514.e1-8. doi: 10.1016/j.ajog.2008.03.010 [published Online First: 2008/05/06]

Figure 1 title: Cohort study participation flow chart

For peer review only - http://bmjopen.bmj.com/site/about/guidelines.xhtml

Figure 1 caption: Data on eligible pregnancies, enrolled pregnancies, refusals, lost to follow-up, and birth outcomes.

For peer review only

For peer review only - http://bmjopen.bmj.com/site/about/guidelines.xhtml

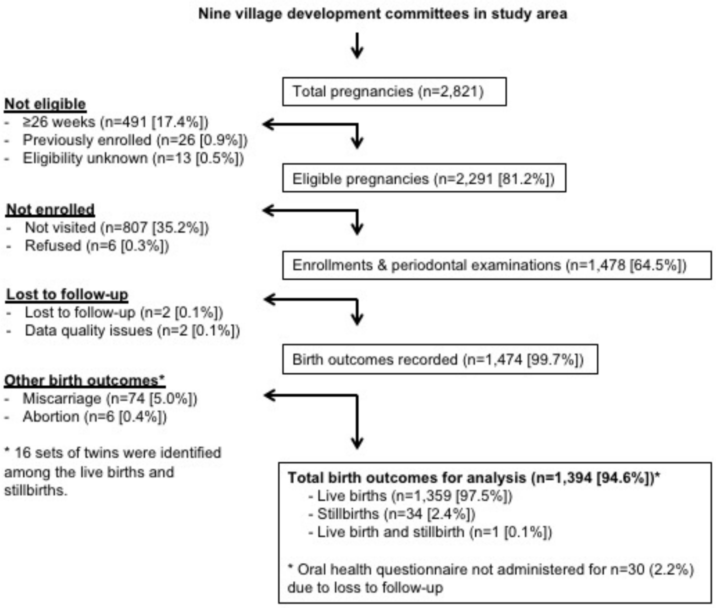

Title: Figure 1: Cohort study participation flow chart

Caption: Data on eligible pregnancies, enrolled pregnancies, refusals, lost to follow-up, and birth outcomes.

254x190mm (72 x 72 DPI)

STROBE Statement—Checklist of items that should be included in reports of ***cohort studies***

	Item No	Recommendation	Page No
**Title and abstract**	1	(*a*) Indicate the study's design with a commonly used term in the title or the abstract<br>(*b*) Provide in the abstract an informative and balanced summary of what was done and what was found	1-3
**Introduction**			
Background/rationale	2	Explain the scientific background and rationale for the investigation being reported	4-5
Objectives	3	State specific objectives, including any prespecified hypotheses	5
**Methods**			
Study design	4	Present key elements of study design early in the paper	6
Setting	5	Describe the setting, locations, and relevant dates, including periods of recruitment, exposure, follow-up, and data collection	6-7
Participants	6	(*a*) Give the eligibility criteria, and the sources and methods of selection of participants. Describe methods of follow-up<br>(*b*) For matched studies, give matching criteria and number of exposed and unexposed	6-9
Variables	7	Clearly define all outcomes, exposures, predictors, potential confounders, and effect modifiers. Give diagnostic criteria, if applicable	7-9
Data sources/ measurement	8*	For each variable of interest, give sources of data and details of methods of assessment (measurement). Describe comparability of assessment methods if there is more than one group	7-9
Bias	9	Describe any efforts to address potential sources of bias	8-9
Study size	10	Explain how the study size was arrived at	8
Quantitative variables	11	Explain how quantitative variables were handled in the analyses. If applicable, describe which groupings were chosen and why	8-9
Statistical methods	12	(*a*) Describe all statistical methods, including those used to control for confounding<br>(*b*) Describe any methods used to examine subgroups and interactions<br>(*c*) Explain how missing data were addressed<br>(*d*) If applicable, explain how loss to follow-up was addressed<br>(*e*) Describe any sensitivity analyses	8-9
**Results**			
Participants	13*	(a) Report numbers of individuals at each stage of study—eg numbers potentially eligible, examined for eligibility, confirmed eligible, included in the study, completing follow-up, and analysed<br>(b) Give reasons for non-participation at each stage<br>(c) Consider use of a flow diagram	10
Descriptive data	14*	(a) Give characteristics of study participants (eg demographic, clinical, social) and information on exposures and potential confounders<br>(b) Indicate number of participants with missing data for each variable of interest<br>(c) Summarise follow-up time (eg, average and total amount)	10-11
Outcome data	15*	Report numbers of outcome events or summary measures over time	10

For peer review only

For peer review only - http://bmjopen.bmj.com/site/about/guidelines.xhtml

Main results	16	(*a*) Give unadjusted estimates and, if applicable, confounder-adjusted estimates and their precision (eg, 95% confidence interval). Make clear which confounders were adjusted for and why they were included<br>(*b*) Report category boundaries when continuous variables were categorized<br>(*c*) If relevant, consider translating estimates of relative risk into absolute risk for a meaningful time period	15-19
Other analyses	17	Report other analyses done—eg analyses of subgroups and interactions, and sensitivity analyses	20-22
**Discussion**			
Key results	18	Summarise key results with reference to study objectives	23
Limitations	19	Discuss limitations of the study, taking into account sources of potential bias or imprecision. Discuss both direction and magnitude of any potential bias	26-27
Interpretation	20	Give a cautious overall interpretation of results considering objectives, limitations, multiplicity of analyses, results from similar studies, and other relevant evidence	27
Generalisability	21	Discuss the generalisability (external validity) of the study results	27
**Other information**			
Funding	22	Give the source of funding and the role of the funders for the present study and, if applicable, for the original study on which the present article is based	28

*Give information separately for exposed and unexposed groups.

Note: An Explanation and Elaboration article discusses each checklist item and gives methodological background and published examples of transparent reporting. The STROBE checklist is best used in conjunction with this article (freely available on the Web sites of PLoS Medicine at http://www.plosmedicine.org/, Annals of Internal Medicine at http://www.annals.org/, and Epidemiology at http://www.epidem.com/). Information on the STROBE Initiative is available at http://www.strobe-statement.org.

For peer review only
